# Intervention effects of traditional Chinese medicine on stem cell therapy of myocardial infarction

**DOI:** 10.3389/fphar.2022.1013740

**Published:** 2022-10-18

**Authors:** Yu Wang, Yuezhen Xue, Hai-dong Guo

**Affiliations:** ^1^ Academy of Integrative Medicine, Shanghai University of Traditional Chinese Medicine, Shanghai, China; ^2^ Institute of Molecular and Cell Biology (IMCB), Agency for Science, Technology and Research (A*STAR), Singapore, Singapore; ^3^ Department of Anatomy, School of Basic Medicine, Shanghai University of Traditional Chinese Medicine, Shanghai, China

**Keywords:** traditional Chinese medicine, stem cells, myocardial infarction, survival, migration

## Abstract

Cardiovascular diseases are the leading cause of global mortality, in which myocardial infarction accounts for 46% of total deaths. Although good progress has been achieved in medication and interventional techniques, a proven method to repair the damaged myocardium has not yet been determined. Stem cell therapy for damaged myocardial repair has evolved into a promising treatment for ischemic heart disease. However, low retention and poor survival of the injected stem cells are the major obstacles to achieving the intended therapeutic effects. Chinese botanical and other natural drug substances are a rich source of effective treatment for various diseases. As such, numerous studies have revealed the role of Chinese medicine in stem cell therapy for myocardial infarction treatment, including promoting proliferation, survival, migration, angiogenesis, and differentiation of stem cells. Here, we discuss the potential and limitations of stem cell therapy, as well as the regulatory mechanism of Chinese medicines underlying stem cell therapy. We focus on the evidence from pre-clinical trials and clinical practices, and based on traditional Chinese medicine theories, we further summarize the mechanisms of Chinese medicine treatment in stem cell therapy by the commonly used prescriptions. Despite the pre-clinical evidence showing that traditional Chinese medicine is helpful in stem cell therapy, there are still some limitations of traditional Chinese medicine therapy. We also systematically assess the detailed experimental design and reliability of included pharmacological research in our review. Strictly controlled animal models with multi-perspective pharmacokinetic profiles and high-grade clinical evidence with multi-disciplinary efforts are highly demanded in the future.

## Introduction

Cardiovascular diseases (CVDs), principally ischemic heart disease (IHD) and stroke, are the leading cause of mortality globally (Roth et al., 2020). Moreover, myocardial infarction (MI) accounts for 46% of all deaths attributed to CVDs (Tsao et al., 2022). MI-induced blood insufficiency leads to myocardial necrosis and fibrotic scar formation, which eventually causes arrhythmias, ventricular dysfunction, and post-infarction congestive heart failure (Henry et al., 2021). Because of the limited capability of myocardial self‐regeneration, the damages are typically irreversible (Tzahor and Poss, 2017). Although good progress has been achieved in medication and interventional techniques such as primary coronary angioplasty, a proven strategy to repair the damaged myocardium has not yet been determined.

Novel methods have been identified for promoting myocardial regeneration by injecting stem cells into the infarcted heart (Miao et al., 2017). Stem cells act as undifferentiated cells, which can divide and differentiate into numerous mature cell types with specialized functions (Bacakova et al., 2018). Stem cells are mainly classified into two groups: embryonic stem cells (ESCs), pluripotent stem cells originating from the internal cellular mass of the blastocyst, and adult stem cells (ASCs) presenting in different tissues throughout the body after development (Lagarkova, 2019). However, most stem cells have defects in the treatment of MI, including low survival rate, immune exclusion, and low differentiation efficiency (Laplane and Solary, 2019). Due to this potential limitation, there has been significant interest in understanding the factors determining the survival of transplanted stem cells (Miao et al., 2017). Moreover, the hostile ischemic environment, rich in inflammation factors; free radicals generated by oxidative stress, and hypoxic areas further limit the efficiency of stem cell-based therapy (Fu et al., 2021).

Overcoming these limitations can improve the efficacy of stem cell therapy for heart diseases. One such strategy is to genetically manipulate the expression of critical genes involved in cell survival. Pro-survival genes such as *Akt*, *Bcl-2*, and *SDF-1* improve stem cell viability after transplantation but with little efficacy (Penn and Mangi, 2008). Other strategies for improving the survival of transplanted stem cells in the ischemic myocardium have been developed, such as genetic modification of transplanted stem cells, stem cell transplantation in combination with growth factor delivery, and cell therapy using various scaffolds (Kim et al., 2013; Afjeh-Dana et al., 2022). A third and more critical strategy for myocardial infarction therapy is to promote angiogenesis and endothelial cell growth in the infarcted heart (Gu et al., 2015). However, only a portion of the stem cells successfully differentiates into cardiomyocytes but distributes in the less ischemic boundary zone. Therefore, transplanted stem cells are unable to form functional cardiac tissues, making the transplantation of stem cells for MI therapy less than optimal.

Chinese botanical and other natural drug substances are a major aspect of traditional Chinese medicine (TCM) and are a rich source of unique chemicals. As such, numerous studies have revealed the role of Chinese medicine in stem cell therapy for MI treatment, including promoting proliferation (Zhu et al., 2017), differentiation (Li et al., 2006), migration (Liu et al., 2013), angiogenesis (Guo et al., 2014), and survival (Cao et al., 2016) of stem cells. Here, we discuss the potential and limitations of stem cell therapy and the regulatory mechanism of Chinese medicines underlying stem cell therapy. We focus on the evidence derived from pre-clinical trials and clinical practices and summarize the theoretical basis for the efficacy of TCM from the perspective of stem cells.

## Potential and limitations of stem cell therapy for treating myocardial infarction

**FIGURE 1:**
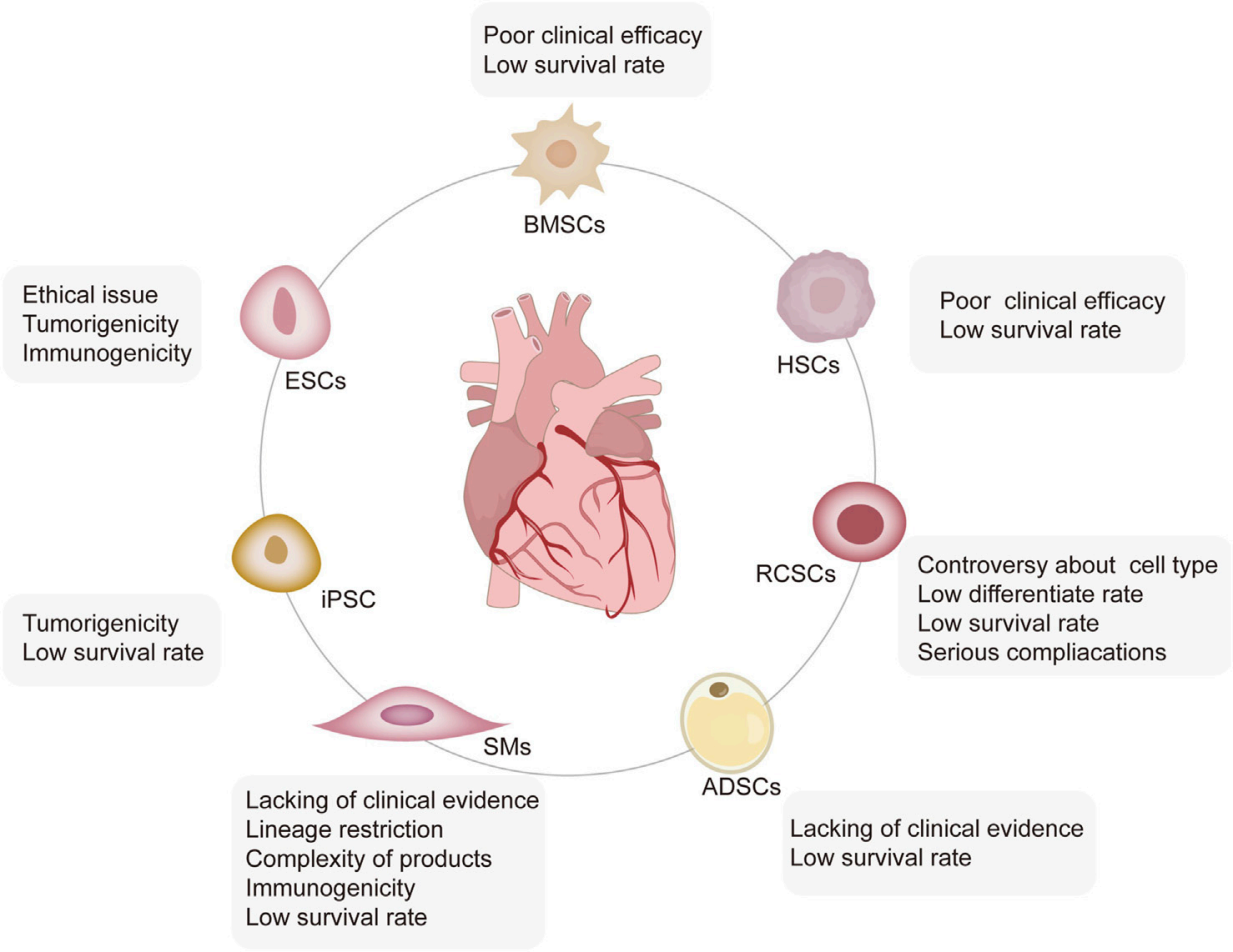
Limitations of stem cell-based therapy. Five types of stem cells, including ESCs, iPSCs, HSCs, RCSCs, ADSCs, and SMs, are involved in MI treatment, each of which has its limitations. ESCs face severe ethical issues with high tumorigenic and immunogenic characteristics; iPSCs have a high rate of tumorigenicity and a low survival rate. BMSCs and HSCs are characterized by self-renewal, no tumorigenicity, low immunogenicity, and strong immunomodulatory ability. However, their low survival rate leads to poor clinical efficacy. The biological functions of the subgroups of RCSCs have not been well detected yet, and the low survival rate, low differentiation rate, and even severe complications limit their application. SMs have lineage restrictions and thus, are unable to produce new cardiomyocytes. Moreover, the complexity, immunogenicity, and low survival of autologous SM products limited their wide application.

Different sources of stem and progenitor cells, including ESCs and ASCs, have been validated for their ability to promote cardiac regeneration and repair (Rigaud et al., 2020). The therapeutic effects of unselected bone marrow cells (BMCs) (Fisher et al., 2014), hematopoietic stem cells (HSCs) (Shafei et al., 2018), mesenchymal stem cells (MSCs) (Ulus et al., 2020), resident cardiac stem cells (RCSCs) (Makkar et al., 2012), and induced pluripotent stem cells (iPSCs) (Drowley et al., 2016) have gained progression in basic translational and clinical applications. Moreover, skeletal myoblasts (SMs) (Menasché et al., 2008) and adipose-derived stem cells (ADSCs) (Davy et al., 2015) constitute other cell populations that may be suitable for cardiac repair. However, each cell category has its practical limitations and translational disadvantages (Figure 1). We discuss these in detail in the following sections.

### Potential and limitations of embryonic stem cells and induced pluripotent stem cell therapy

Although ESCs possess some distinctive advantages in cardiac repair, including their pluripotency, which means that they can differentiate into all types of cells, there are still ethical and regulatory concerns (Lo and Parham, 2009; Khan et al., 2015). More importantly, the risk of malignancies further limits ESC-based treatments (Madigan and Atoui, 2018). Although some methods to inhibit tumorigenesis have been used, reliable approaches to modulate and control differentiation in a controllable and efficient manner are still scarce (Carvalho et al., 2015). In addition, ESCs trigger severe immune rejection following allogeneic application (Samak and Hinkel, 2019). iPSCs are a promising alternative to ESCs in regenerative medicine, which can be redifferentiated from adult somatic cells (e.g., fibroblasts, epidermal cells, and hemocytes) using reprogramming techniques (Hynes et al., 2015). iPSC technology has been developed for auto-transplantation, bypassing ethical concerns associated with destroying fertilized embryos, and without an immune response (Faiella and Atoui, 2016). Moreover, unlike ASCs, which partly differentiate into a limited number of cell types, the iPSCs have a great potential to give rise to all lineages of cells (Chamberlain, 2016). While iPSCs give full play to the advantage of ESCs and ASCs, safety issues with these cells need to be addressed before they can be used in clinical settings. The property of infinite proliferation in iPSCs is a double-edged sword because if cells keep proliferating even after transplantation, they may result in tumors (Malchenko et al., 2014). Therefore, finding the best source of stem cells has always been one of the main problems in this field.

### Potential and limitations of bone marrow mesenchymal stem cell therapy

One of the most promising cardiac cell-based therapies is unselected BMCs therapy, which has clinical surveillance for up to 5 years (Fisher et al., 2018). BMCs have some advantages for clinical application, including the ease of procurement and harvesting *ex vivo*, a sufficient number, and purity, and both have the properties of stem and progenitor cells (Haider, 2018). However, the results of clinical trials suggested that the effectiveness of BMCs is usually modest and less than the expectations of the originally intended result (Wollert et al., 2017; Zhang et al., 2021). In a randomized clinical trial, BMC intracoronary transplantation in acute myocardial infarction (AMI) patients did not increase the left ventricular ejection fraction (LVEF), and only a slight improvement in myocardial perfusion was observed in the BMC group (Grajek et al., 2010).

Therefore, more studies focused on the different subgroups of BMCs, which are divided into two populations, HSCs and non-HSCs.

### Potential and limitations of hematopoietic stem cell therapy

As for HSCs, markers of CD133 and CD34 are generally adopted to select specific cell populations, and CD34^+^ cells possess more endothelial lineage-phenotype cells than CD133^+^ cells, considered “early” endothelial progenitor cells (EPCs) in HSCs (Chen et al., 2021a). A randomized controlled trial compared the LVEF after MI between unsorted and CD34^+^/CXCR4^+^-sorted BMCs (Tendera et al., 2009). After 6 months, a 3% increase in the LVEF was observed in patients treated, as discussed previously, whereas the control group remained unchanged. The CARDIO133 phase III clinical trial was designed to evaluate the effect of intra-myocardial injection of CD133^+^-sorted BMCs in cardiac repair (Nasseri et al., 2014). The results showed that CD133^+^-sorted BMCs improved regional scar perfusion but did not affect LV function. Presumably, this occurred because of the low baseline levels of HSCs, which limit their efficacy. Taken together, despite the diversity and therapeutic potential of BMC-based therapy, the clinical response of this therapy leaves great room for improvement; incorporating different combinations of biomarkers to reinforce the cellular repair capacity needs to be studied in the future.

### Potential and limitations of mesenchymal stem cell therapy

MSCs, non-HSCs in bone marrow or adipose tissue, represent another potential selection for stem cell-based therapy. MSCs play an essential role in MI therapy because of their unique properties, including the ability to differentiate into cardiomyocytes (although controversial) (Wang et al., 2015), immunomodulatory property (Eldaly et al., 2022), anti-fibrotic activity (Li et al., 2015), and promotion of angiogenesis (Gao et al., 2017). Regarding differentiation ability, the combined treatment of MSCs and exogenous Jagged1 activated Notch1 signaling and caused multilineage differentiation (Ding et al., 2015). Moreover, the overexpression of miRNA1-2 in mouse MSCs promotes the differentiation of MSCs into cardiomyocyte-like cells through activation of the Wnt/β-catenin signaling pathway (Shen et al., 2017). However, it is widely acknowledged that the central effect of MSCs in the treatment of MI relies on the paracrine effect and not on the differentiation of MSCs into cardiomyocytes (Guo et al., 2020).

Bone marrow-derived MSCs (BMSCs), one of the adult pluripotent stem cells with great differentiation potential, low immunogenicity, and immune regulatory abilities, regulate different pathways of immune cells in a paracrine way (Bulati et al., 2020). BMSCs have great clinical application value and broad prospects due to their characteristics. However, the therapeutic efficacy of BMSC-based therapy *in vivo* remains a challenge. Previous studies have consistently indicated the poor survival of BMSCs after transplantation; about 90% of BMSCs died within the first 4 days (Zhao et al., 2019). Some researchers showed that BMSCs have a low survival rate in the cardiac environment, and most transplanted cells may disappear soon after transplantation (van der Spoel et al., 2011; Blocki et al., 2015; Li et al., 2016). When BMSCs are transplanted to ischemic zones, a hostile cardiac microenvironment, with a major proportion of reactive oxygen species (ROS), hypoxia, inflammation, fibrosis, and oxidative stress limit their survival potential (Lin et al., 2020). Currently, autologous and allogeneic BMSC transplantations are under investigation, whereas their therapeutic efficacy remains uncertain. In a clinical trial, 69 patients with AMI who underwent successful percutaneous coronary intervention (PCI) were transferred to receive an intracoronary infusion of BMSCs and saline. The results showed that BMSCs, at least in part, improved cardiac function without deaths (Chen et al., 2004). In another randomized, controlled trial, patients with ST-elevation AMI after reperfusion treatment within 12 h were randomly divided into BMSC-injection or standard medical treatment groups (Gao et al., 2013). BMSC-based treatment improved cardiac function and myocardial viability within the infarct area after 6 months in both groups compared with baseline; however, no significant difference was evident between these groups. The clinical benefits of BMSC-based therapy in patients with MI need further investigation and re-evaluation.

Moreover, adipose tissue also serves as a source of MSCs named ADSCs. Accumulating studies suggest that the effects of ADSCs are majorly related to paracrine action rather than trans-differentiation. The exosomes isolated from ADSCs attenuated cardiac injury after MI by activating the S1P/SK1/S1PR1 signaling pathway and increasing macrophage transition to the M2 phenotype (Deng et al., 2019). It was reported that conditional medium (CM) containing miR-221/222 from ADSCs significantly reduced cardiac apoptosis and fibrosis by reducing PUMA and ETS-1 expression, respectively (Lee et al., 2021). Meanwhile, miR-93-5p-encapsulating exosomes from ADSCs protected the myocardium by inhibiting autophagy and inflammatory response (Liu et al., 2018). Moreover, ADSCs-SIRT1-exosomes can recruit EPCs to the infarct area through Nrf2/CXCL12/CXCR7 signaling (Huang et al., 2020). However, clinical evidence remains scarce.

### Potential and limitations of resident cardiac stem cell therapy

The adult mammalian heart has traditionally been thought of as a terminally differentiated organ, and cardiomyocytes have limited ability to regenerate for a long time (Windmueller et al., 2020). Nevertheless, RCSCs have been found and isolated in adult mammalian hearts, with multiple phenotypes, and they exhibit self-renewal capacity and multilineage potential, including differentiation into cardiomyocytes, smooth muscle cells, and endothelial cells under suitable conditions (Valiente-Alandi et al., 2016). Indeed, they are an appropriate candidate for cardiac regeneration therapy, for they are intrinsically programmed to form cardiac tissues and differentiate into parenchymal cells and coronary vessels rapidly upon activation (Uygur and Lee, 2016). Multiple subtypes of RCSCs are classified through surface markers and transcription genes, including c-kit^+^ RCSCs, Sca-1^+^ RCSCs, Islet-1^+^ RCSCs, side population RCSCs, and cardiosphere-derived CSCs (Li et al., 2019a). However, until now, whether RCSCs population extracted based on markers are of different types or whether they present a co-primitive cell type as the originator of these cells remains unclear. Moreover, the biological functions of the subgroups of RCSCs have not been well detected yet. There are still tremendous controversies about whether RCSCs can differentiate into a functional myocardium, especially after the retracted studies of Piero Anversa (Beltrami et al., 2003). It was reported that although c-kit^+^ RCSCs cannot differentiate into new cardiomyocytes directly, they can improve heart function through an immune response by recruiting the accumulation of CCR2^+^ and CX3CR1^+^ macrophages (Vagnozzi et al., 2020). Challenges still remain regarding the future direction of RCSCs before bringing them into clinical practice. For instance, difficulty in autogenous cell isolation and the low survival rate of RCSCs in infarcted hearts limited their application. Moreover, numerous complications have been discussed after RCSC implantation (Eschenhagen et al., 2017). It is essential to explore novel methods to improve homing, survival, proliferation, and differentiation of RCSCs in injured hearts.

### Potential and limitations of skeletal myoblast therapy

SMs are commonly isolated from muscle tissues and suffer *ex vivo* expansion for MI treatment. Pre-clinical evidence has proved their repair effects in MI (Imanishi et al., 2011). Furthermore, some Phase I clinical trials have generated exciting results for the therapeutic efficiency of SMs in MI, suggesting an increasingly global and regional LVEF, and improvement in cardiac tissue viability in the infarct areas (Herreros et al., 2003; Siminiak et al., 2004; Dib et al., 2009). However, the results of the Phase II MAGIC trial showed that myoblast injections in patients with depressed LV function failed to improve their heart function and increased the number of early postoperative arrhythmic events (Menasché et al., 2008). The other limitations of SMs are summarized as follows. First, the most severe drawback of SM-based therapy may be their lineage restriction and inability to produce new cardiomyocytes (Terajima et al., 2014). Second, the complexity of autologous SM products limits their wide application (Ponsuksili et al., 2022). Third, the immunogenicity of SMs increases the risk and complications in clinical treatments. Lastly, SMs also have a low survival rate; up to 90% of SMs die over the first days after engraftment, and the myoblast-transplanted human heart confirms the scarcity of persisting myotubes in scar tissues (Skuk and Tremblay, 2019).

## Regulatory mechanism of Chinese medicines underlying stem cell therapy

**FIGURE 2:**
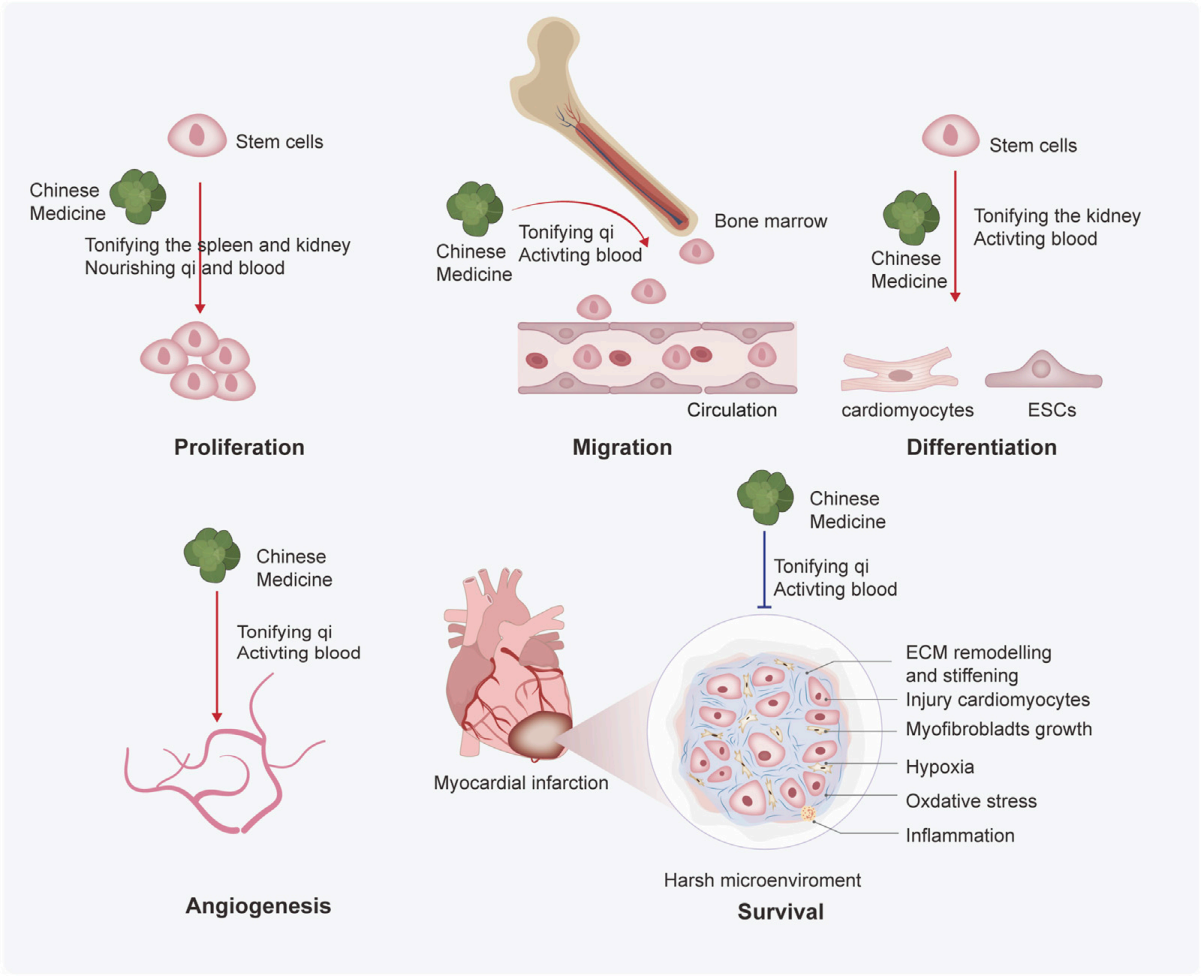
Mechanisms of Chinese medicines based on TCM theory enhance the efficacy of stem cell therapy. Chinese medicine promotes the proliferation, migration, differentiation, angiogenesis, and survival of stem cells. Through the summary analysis from commonly used prescriptions, the mechanisms based on TCM theory may be finally inferred as follows: 1) Chinese medicine promotes the proliferation of stem cells by tonifying the spleen and kidneys, nourishing qi and blood; 2) Chinese medicine promotes migration of stem cells by tonifying the qi and activating blood; 3) Chinese medicine promotes differentiation of stem cells by tonifying the kidney and activating blood; and ) Chinese medicine promotes angiogenesis and survival by tonifying qi and activating blood.

To overcome the limitations of stem cell therapy, researchers have applied various methods to find approaches to enhance its efficacy for MI. Therein, Chinese medicines stand out due to their high efficiency and low toxicity, offering a feasible approach to compensate for the disadvantages of stem cell therapy. Based on TCM theory, Zhang et al. studied the related research about stem cells and kidney essence, found their similarity in the origin of life and physiological function, and provided new ideas for the research on the basic theory of TCM (Zhang and Zhang, 2018). Moreover, they further refined the view of “kidney properties” activating blood and removing stasis and clarified that stem cells were the material basis of “kidney properties” (Zhang et al., 2016). Next, we discuss how Chinese medicines improve the efficacy of stem cell therapy in TCM theories. More importantly, we will categorize TCM in prescription, botanical and other natural drug substances, pure compounds; and experiments *in vivo* and *in vitro* to better understand the mechanisms of TCM treatment in MI. We have summarized the mechanism of Chinese medicines for stem cell therapy in Figure 2 and Table 1, 2.

**TABLE 1:** Summary of prescriptions on stem cell therapy.

Prescriptions	Dose & Duration	Experimental subject	Effect
Guanxin Danshen formulation (冠心丹参方)^a^	100 mg/kg/d, 28 d	SD male rats	Promoting survival of BMSCs (Han et al., 2019)
Taohong Siwu decoction (桃红四物汤)^a^	1.13 g/ml/250 g/d (concentrated extraction), 28 d	SD male rats	Decreasing mitochondrial fission (Luo et al., 2019)
Xuefu Zhuyu decoction (血府逐瘀汤)^a^	5%, 10%, and 15%-drug serum	rEPC	Induced angiogenesis through EPC activation (Gao et al., 2010)
Shuangxinfang (双心方)^a^	1 ml/100 g, 3, 7, and 14 d	Male SD rats	Promoting mobilization of BMSCs (Wang et al., 2019)
Shuanglong formula (双龙方)^a^	1 μg/ml, 24 h	rMSCs	Promoting differentiation of BMSCs (Fan et al., 2010)
Tongxinluo (通心络)^a^	50 mg/kg/d, 7 d	Chinese mini pigs	Promoting survival and differentiation of MSCs (Qian et al., 2007)
	50–400 μg/ml, 6 h	rMSCs	Inhibiting apoptosis of MSCs (Li et al., 2014)
	400 μg/ml (pre-treat), 24 h	SD rats	Promoting cardiac repair combined with Exo (Xiong et al., 2022a; Xiong et al., 2022b)
Danhong injection (丹红注射液)^a^	1.5 ml/kg/d, 28 d (ip)	Male C57BL/6J mice	Promoting mobilization and angiogenesis of BMSCs (Chen et al., 2018)
Xuesaitong injection (血塞通注射液)^a^	150 mg/kg/d, 1, 7, and 14 d (ip)	Female and male Wistar rats	Promoting mobilization of BMSCs (Zhang et al., 2011)
Si–Wu decoction (四物汤)^a^	0.03–0.3 mg/ml, 72 h	rMSCs	Promoting proliferation of BMSCs (Zeng et al., 2008)
Gu Ben Pei Yuan San (固本培元散)^a^	5% and 10% drug powder mixed in feed for 7 d, 1 m, and 2 m	Male C57BL/6J mice	Promoting differentiation of iPSCs (Cui et al., 2019)

^a^ The compositions of the prescriptions are presented in Supplementary Table S1.

**TABLE 2:** Summary of natural drug substances on stem cell therapy.

Botanical and other natural drug substances	Compounds and metabolite	Dose and duration	Experimental subject	Effect
*Astragalus mongholicus* Bunge (Huang Qi)	Astragaloside IV	0.1–2 μg/ml (pre-treat), 72 h	SD rats; mMSCs	Promoting mobilization of BMSCs (Xie et al., 2013)
		20–50 mg/kg/d, 14 d; 10–160 μmol/L, 72 h	SD rats; rHUVECs	Promoting angiogenesis (Cheng et al., 2019)
	Astragaloside	5–500 ng/ml, 24–48 h	rMSCs	Promoting proliferation of MSCs (Zhu et al., 2017)
*Epimedium brevicornu* Maxim. (Yin Yang Huo)	Icariin	20–320 μg/L, 72 h	rBMSCs	Promoting proliferation of BMSCs *via* activating ERK and p38 MAPK (Qin et al., 2015)
Plastrum testudinis (PT, Gui Ban)	Fatty acid, fatty acid esters, and steroid	0.01–3 mg/ml, 24 h-5 d	rMSCs	Promoting proliferation of BMSCs (Chen et al., 2007)
	Fatty acid, fatty acid esters, and steroid	0.03–3 mg/ml, 24 h-5 d	rMSCs	Promoting proliferation of BMSCs (Wang et al., 2012a)
	Cholesterol myristate	30–300 μg/ml 72 h, 1 d, 3 d	rMSCs	Promoting proliferation of BMSCs (Chen et al., 2009)
*Rehmannia glutinosa* (Gaertn.) DC. (Di Huang)	Rehmannia glutinosa oligosaccharide (RGO)	1–400 mg/L, 72 h	hADMSCs	Promoting survival of ADSCs (Zhang et al., 2012)
*Codonopsis pilosula* (Franch.) Nannf. (Dang Shen)	—	10 mg/300 g, 10 d; 0.5 mg/ml, 10 d	Male Wistar rats; ES	Promoting differentiation of ESCs (Wang et al., 2021)
*Panax ginseng* C.A.Mey. (Ren Shen)	Ginsenosides Re	135 mg/kg, 28 d	Male Wistar rats	Inhibiting fibrosis (Yu et al., 2020)
*Ligusticum striatum* DC. (Chuan Xiong); *Paeonia lactiflora* Pall. (Shao Yao)	—	55 mg/kg/d, 21 d	Male C57BL/6J mice	Promoting mobilization and angiogenesis of BMSCs (Shi et al., 2019)
*Paeonia lactiflora* Pall. (Shao Yao)	Total paeony glucosides (TPGs)	5–40 μg/ml, 24 h	rH9c2	Preserving antioxidant defense (Luo et al., 2013)
*Geum japonicum* var. chinense F.Bolle	The angiogenic and cardiomyogenic fractions were mixed as myocardial repair fraction	0.3 mg, 30 d; 20–80 μg/ml, 72 h	SD rats; HCAEC	Promoting angiogenesis and cardiomyogenesis (Li et al., 2006)
	Cardiogenin	0.3 mg/kg/d, 14 d; 10 μg/ml, 4 d	SD rats; rMSCs	Promoting differentiation of BMSCs (Cheng et al., 2009)
		2 mg/kg/d, 14 d	SD rats	Promoting differentiation of BMSCs (Lin et al., 2012)
*Salvia miltiorrhiza* Bunge (Dan Shen)	Salvianolic acid B	10 μmol/L, 28 d; 1–100 μmol/L 24 h	Female SD rats; rMSCs	Promoting proliferation, differentiation, and angiogenesis of BMSCs (Guo et al., 2014)
		80, 160 mg/kg, 30 d; 5–20 ng/ml, 24 h	Male KM mice; CFs	Inhibiting fibrosis (Gao et al., 2019)
	Tanshinone IIA	0.1–2 μg/ml, 72 h	SD rats; mMSCs	Promoting mobilization of BMSCs (Xie et al., 2013)
		0.1–2 μg/ml, 72 h		
		1.5 mg/kg/d, 28 d; 10 μM, 24 h	SD rats; rCFs	Inhibiting fibrosis (Chen et al., 2021b)
*Panax notoginseng* (Burkill) F.H. Chen (San Qi)	Notoginsenoside R1	267 ng/kg (nanoparticle), 48 h 0.1–1,000 μg/ml, 2 h	BALB/c nude mice, C57BL/6 mice; H9C2, rCMs	Improving cardiac function (Li et al., 2022)
	Total panax notoginsenosides	0.1–100 μg/ml, 10 d	rBMSCs	Promoting angiogenesis of BMSCs (Zheng et al., 2013)
*Scutellaria baicalensis* Georgi (Huang Qin)	Baicalin	5–500 ng/ml, 24–48 h	rMSCs	Promoting proliferation of MSCs (Zhu et al., 2017)
**/**	Resveratrol	25 mg/kg/day, 28 d	Male C57BL6J mice	Promoting mobilization of BMSCs (Hong et al., 2015)
/	Hexadecanoic acid	3–30 μg/ml, 72 h	rMSCs	Promoting proliferation of BMSCs (Chen et al., 2010)

### Chinese medicines promote the proliferation of stem cells by tonifying the spleen and kidney, nourishing qi and blood

The regenerative potential of stem cells is directly proportional to the number of available stem cells and their proliferation ability (Hedderich et al., 2020). Among these stem cells, BMSCs can differentiate into various lineages without risk of immunological rejection, so they have been mostly applied in stem cell transplantation. With developments in chemical purification technologies and mass spectrometry, the active compounds of numerous Chinese medicine formulas have been successfully purified, and their effects on BMSC proliferation have been detected.

For instance, Si–Wu decoction (SDE), a classic blood-tonifying formula in TCM, has been adopted for clinical treatment in China for centuries. Zeng H. P. et al*.*, extracted the active ingredients of this formula using ethyl acetate/chloroform to explore its proliferation-promoting effects on BMSCs (Zeng et al., 2008). A total of 20 compounds were obtained, and ligustilide displayed the best proliferation-promoting effect. Palmitic acid methyl ester and stearic acid ethyl were also responsible for promoting the proliferation of BMSCs. Extractions with 0.3 mg/ml concentration had a better efficacy of BMSCs proliferation than bFGF, a common positive control in this area. Furthermore, another constituent of flavonoids from the *Epimedium brevicornu* Maxim. [Berberidaceae] (EBM), icariin (ICA), also facilitated the proliferation of BMSCs by activating ERK and p38 MAPK signaling pathways, and regulating their downstream transcription factors Elk1 and c-Myc (Qin et al., 2015). Several studies found the active components of Plastrum testudinis [Testudinidae] (PT) were able to promote BMSCs proliferation (Chen et al., 2007; Wang et al., 2012a). Further mechanism studies suggested that myristate is the main active component of PT, and it can increase the release of bone morphogenetic protein 4 (BMP4) from BMSCs in a time- and dose-dependent manner (Chen et al., 2009). Buzhong Yiqi decoction (BYD), a tonic formula of TCM, and its active compound, hexadecanoic acid (HA), was responsible for promoting the proliferation of BMSCs (Chen et al., 2010). Moreover, astragaloside, a compound from qi-replenishing Chinese medicine, promoted differentiation and proliferation, inhibited apoptosis, and reduced the inflammatory effects of BMSCs (Zhu et al., 2017).

ADSCs are believed to be a suitable cell source of regenerative treatment for their self-renewal capacity and multilineage differentiation. Chinese medicine also exhibits proliferation-promoting action in ADSCs. Rehmannia glutinosa oligosaccharide (RGO), an extract from Chinese medicine, has been proven to increase proliferation and relieve H_2_O_2_-induced apoptosis of ADSCs by the paracrine secretion of VEGF and HGF (Zhang et al., 2012). These studies have now allowed for refinement in the understanding of TCM with respect to pharmacological regulation of proliferation of stem cells and may be helpful to stem cell biology and therapy.

In summary, according to the TCM theory—“Kidney dominated bone marrow” (Gu et al., 2019), Chinese medicines used in promoting the proliferation of stem cells are mainly kidney-tonifying medicines such as ICA and PT or blood-nourishing medicines such as ligustilide and RGO. To a certain extent, this conforms to the mechanism of the inadequate number of stem cells in modern medicine and provides an integrative theoretical foundation for the proliferation of stem cells.

### Chinese medicines promote the mobilization and migration of stem cells by tonifying the qi and activating blood

Stem cells need to be recruited to infarct areas after reaching a certain number. TCM promotes the mobilization and migration of stem cells as well. Several BMSC-mobilizing factors, including transforming growth factor-β (TGF-β), tumor necrosis factor-α (TNF-α), stromal-derived factor-1 (SDF-1), and hepatocyte growth factor (HGF), can promote the migration of BMSCs and induce cardiac repair (Yu et al., 2015; Sun et al., 2018). Guanxin Danshen (GXDS) formulation with the preparation of dripping pills, which mainly activates blood in various diseases, increased SDF-1 levels in the infarcted area and enhanced the migration of BMSCs (Han et al., 2019). Shuangxinfang aqueous extract (Wang et al., 2019) and classical TCM prescriptions derived from the Danshen decoction and Baihe Dihuang decoction promoted the mobilization of BMSCs, inhibited the inflammatory response, and improved heart function after AMI. As a new dosage form of TCM, TCM injection (TCMI) is an important step in the modernizing of TCM. Danhong injection (DHI) increased the residence of BMSCs in cardiac tissue by regulating the SDF1/CXCR4 signaling (Chen et al., 2018). Moreover, the aqueous extract of *Ligusticum striatum* DC. [Apiaceae] and *Paeonia lactiflora* Pall. [Paeoniaceae] protected cardiomyocytes by promoting angiogenesis and mobilization of stem cells (Shi et al., 2019). Consequently, TCM prescriptions for promoting blood circulation or removing blood stasis are useful in promoting the migration of stem cells and the treatment of MI. Meanwhile, the specific blood-activating compounds are investigated as follows.

Tanshinone IIA (TIIA)- and astragaloside IV-stimulated BMSCs showed enhanced capacities of homing to ischemic myocardium partially by upregulation of the CXCR4 expression (Xie et al., 2013). Panax notoginseng saponins (PNS) combined with G-CSF to promote c-kit^+^ BMSCs from the marrow into blood circulation and mobilized their “homing” to the infarction sites (Zhang et al., 2011). Further studies revealed that PNS increased the mobilization of progenitor cells *via* SDF-1α-CXCR4 interaction, thus decreasing the sizes of atherosclerotic plaques (Liu et al., 2013) as well as resveratrol (RSV) (Hong et al., 2015). Additionally, acupuncture is an important part of TCM and plays an essential role in stem cell mobilization as well. Recently, we have demonstrated that electro-acupuncture can repair myocardial damage by regulating the SDF-1/CXCR4 axis (Zhao et al., 2022).

### Chinese medicines promote the differentiation of stem cells by tonifying the kidney and activating blood

The most important suggested mechanism of stem cell therapy is a substitution of injured cells by brand new stem cells, which needs successful differentiation in infarct areas (Müller et al., 2018). Nevertheless, in all cases, the differentiation efficiency of stem cells is low, limiting the progression of stem cell differentiation in stem cell therapy (Bian et al., 2019). Due to its importance, TCM has been widely used for the differentiation of stem cells.

For example, *Geum japonicum* var. Chinense F. Bolle [Rosaceae] (GJ), usually used in the Miao ethnic minority group, promoted the cardiogenic differentiation capability of BMSCs, thus repairing infarcted hearts (Cheng et al., 2009). Further studies have shown that cardiogenin is the main active compound of GJ, which stimulates the processes of angiogenesis and cardiomyogenesis (Li et al., 2006; Lin et al., 2012). The Shuanglong formula (SLF) composed of ginsenosides Rg1 and salvianolic acid B (SalB) promoted BMSCs into cardiomyocyte-like cells (Fan et al., 2010). Therein, SalB (Guo et al., 2014), a water-soluble component of *Salvia miltiorrhiza* Bunge [Lamiaceae] induced BMSCs to differentiate into vascular endothelial cells (VECs), but not cardiomyocytes, improving angiogenesis and heart function after BMSC transplantation mainly through a paracrine effect. Long-term oral intake of Gu Ben Pei Yuan San (GBPYS) powder significantly improved cardiac function by promoting the division of both cardiomyocytes and iPSC-derived cardiomyocytes *in vitro*. Oral intake of GBPYS improved heart repair after myocardial damage in adult mice (Cui et al., 2019). GBPYS feeding for 3 months had no apparent toxicity to the liver, kidneys, and blood in normal mice, suggesting the relative safety of TCM treatment. Moreover, the extracts from *Codonopsis pilosula* (Franch.) Nannf. [Campanulaceae] promoted the cardiogenic differentiation of ESCs (Wang et al., 2021).

In conclusion, replenishing qi and activating blood is the basic therapeutic principle of TCM in regulating stem cell differentiation. In addition, electrical stimulation enhanced the efficiency of cardiac differentiation into iPSCs and promoted cardiomyocyte maturation (Ma et al., 2018). In fact, ESCs and iPSCs can differentiate into spontaneously beating cardiomyocytes, while ASCs can only be differentiated into cardiac cell types of expression of cardiomyocytic markers (Gurusamy et al., 2018). However, small molecular compounds facilitate the trans-differentiation of fibroblasts into cardiomyocytes directly (Fu et al., 2015) or induced ASCs to iPSCs (Guan et al., 2022). Thus, identifying potential natural drug compounds may provide new methods for developing regenerative therapeutic strategies.

### Chinese medicines promote angiogenesis of stem cells by tonifying qi and activating blood

MI inflicts massive damage to the coronary micro-circulation, resulting in vascular disintegration and rarefication of capillaries in the ischemia area (Wu et al., 2021). Cardiac repair after MI involves complex angiogenesis, which starts in the infarct border region and expands to the infarct core. TCM facilitates angiogenesis in the infarct zone through several mechanisms.

EPCs serve as endogenous repair cells to counteract endothelial cell damage, substitute dysfunctional endothelium, and repair tissue after MI (Berger et al., 2013). Xuefu Zhuyu decoction (XFZYD) induced the angiogenesis of EPCs and promoted capillary tube formation (Gao et al., 2010). BMSCs have a strong ability to promote angiogenesis, and TCM combined with them to enhance the process of angiogenesis. DHI (Chen et al., 2018) and PNS (Zheng et al., 2013) increased the expression of VEGF-A of BMSCs in the marginal zone of infarction. The combination of *Ligusticum striatum* DC. [Apiaceae], *Paeonia lactiflora* Pall. [Paeoniaceae] (Shi et al., 2019), and SalB (Guo et al., 2014) protected the ischemic myocardium through angiogenesis. Astragaloside IV (AS-IV) promoted angiogenesis and cardio-protection after MI by activating the PTEN/PI3K/Akt signaling pathway (Cheng et al., 2019).

### Chinese medicines promote the survival of stem cells under the cardiac microenvironment by tonifying qi and activating blood

The efficacy of stem cell-based therapy is based on the survival of stem cells, as well as on the alteration of phenotype and biology that may take place on these cells after engraftment (Franchi et al., 2020). The post-ischemic myocardial microenvironment, characterized by inflammation, oxidative stress, hypoxia, and fibrosis, may inhibit the survival of stem cells (Wei et al., 2016). TCM can protect stem cells by countering the hostile cardiac microenvironment.

Tongxinluo (TXL) is extracted and concentrated from a group of botanical and other natural drug substances, including *Panax ginseng* C.A. Mey. [Araliaceae], *Paeonia lactiflora* Pall. [Paeoniaceae], *Cinnamomum camphora* (L.), J. Presl [Lauraceae], and Ziziphus jujuba Mill. [Rhamnaceae], which benefits qi and performs the function of blood activation (Qi et al., 2015). It could induce the survival and differentiation of BMSCs through the inhibition of apoptosis, oxidative stress, less fibrosis, and inflammatory cell infiltration with more surviving myocardium (Qian et al., 2007; Li et al., 2014; Xiong et al., 2022a). Moreover, TXL-pretreated BMSCs significantly improved cardiac repair through the exosomal transfer of miR-146a-5p by the IRAK1/NF-κB p65 pathway, which may have the potential for clinical translation (Xiong et al., 2022b). In addition, the GXDS formulation increased the number of injected BMSCs in the infarct area by decreasing cell apoptosis and promoting angiogenesis in the peri-infarction and infarction area (Han et al., 2019). Total paeony glucosides (TPGs) extracted from the roots of *Paeonia lactiflora* Pall. [Paeoniaceae] alleviated the dysfunction of cardiomyoblast by preserving antioxidant defense (Luo et al., 2013). Moreover, SalB (Gao et al., 2019) and ginsenoside Re (Yu et al., 2020) inhibited the fibrosis process of the myocardial *via* regulating TGF-β/Smads signal pathways, whereas tanshinone IIA showed anti-fibrosis action by inhibiting oxidative stress (Chen et al., 2021b). Tetramethylpyrazine/ligustrazine (TMP) increased the survival rate of ADSCs, probably inducing the expression of transcription factors associated with fat formation, including peroxisome proliferator-activated receptor γ (PPARγ), CCAAT/enhancer-binding protein α, and Alu (Zhou et al., 2020). Our previous work has also shown that Taohong Siwu decoction (THSWD) aqueous extract improved the local ischemic microenvironment by decreasing mitochondrial fission after MI (Luo et al., 2019). Moreover, we designed a nanoparticle of MSN-Notoginsenoside R1 (NGR1)-CD11 b antibody, which enhanced the targeting of NGR1 *via* activation of AKT and MAPK signaling pathways and might provide a new method for targeted drug delivery systems for the MI (Li et al., 2022). Taken together, TCM promotes the survival of stem cells by ameliorating hostile microenvironment in infarction areas, including remodeling inflammation microenvironment, fibrosis microenvironment, hypoxia microenvironment, oxidative stress microenvironment, and angiogenesis microenvironment.

## Deficiencies of Chinese medicines in stem cell therapy

Although the pre-clinical evidence shows that TCM is helpful in stem cell therapy, further mechanisms involved in TCM have not been thoroughly investigated. We systematically assessed the detailed experimental design and reliability of included pharmacological research in our review according to the consensus of the best practice in research (Heinrich et al., 2020). Of the total 37 MI studies, 10 *in vitro,* 12 *in vivo*, and 15 *in vivo* and *in vitro* studies, *only* one *study* provided patient-relevant results. Among these 37 studies, only one study evaluated the toxicity of TCM (Cui et al., 2019), and three studies used positive control (Zeng et al., 2008; Zhang et al., 2011; Gao et al., 2019). Overall, the majority of the studies present specific experimental details and verify the effectiveness of TCM treatment. However, the putative TCM efficacy based on pre-clinical studies may not be accurate and comprehensive enough.

Pharmacokinetic (PK) studies are essential to build concentration-activity/toxicity and promote target identification of Chinese medicine (Yan et al., 2018). TCM PK routines include five dimensions: 1) system analysis of chemical substances using liquid chromatograph mass spectrometry (LC–MS) together with the utilization of data in available chemical databases; 2) identification of the absorbed prototypes, absorbed metabolites, and unabsorbed constituents of TCM *in vivo*; 3) comprehensive study of the therapeutic mechanisms of TCM; 4) establishment of the qualitative and quantitative pharmacokinetics–pharmacodynamics (PK–PD) patterns by multidimensional data and mathematical modeling; and 5) validation of the main compounds and targets by gene-editing technology (Xu et al., 2021). Most studies in this review have met the basic requirements of PK studies. For example, silica gel column chromatography was used to identify the extraction of Si–Wu decoction (Zeng et al., 2008), and GC–MS was adopted to analyze the active compounds of Plastrum testudinis [Testudinidae] (Chen et al., 2007) and Buzhong Yiqi decoction (Chen et al., 2010). However, the plasma drug PK involves absorption, distribution, metabolism, and excretion of TCM in the body which has not been directly examined in these studies. TCM may perform a synergistic function in treating MI in combination with stem cell-based therapy by regulating the multi-targets through various signaling pathways. Strictly controlled animal models with multi-perspective pharmacokinetic evaluation need urgent investigation.

In addition, the efficacy of TCM in stem cell therapy still lacks high-grade clinical evidence. Zhang et al*.* (2020) systematically reviewed the effect of TCM on patients with MI, but the evidence from clinical trials was insufficient to assess the effect of TCM on patients with MI. Further rigorously designed random clinical trials with a large cohort of patients are required to verify or discover the efficacy of TCM in treating MI. Multi-disciplinary efforts are highly demanded to translate TCM-based treatment into a more persuasive proof of clinical efficacy.

Moreover, the side effects of TCM in stem cell therapy cannot be ignored. SMB, a commonly used botanical drug for MI treatment, is considered relatively safe and well tolerated during the treatment (Wang et al., 2012b). However, SMB injection may cause body weight loss and even increase the total bilirubin level and focal inflammation in a dose-dependent manner. In a cohort study, 30,180 patients were recruited to evaluate the adverse events of SMB, and the results showed that SMB might cause rashes, pruritus, platelet count abnormalities, and palpitations (Jia et al., 2019). Nevertheless, the most adverse events of SM were mild to moderate and cleared up after SM treatment withdrawal. *Carthamus tinctorius* L. [Asteraceae], with the efficacy of activating blood and resolving stasis, led to acute liver failure (ALF) in a few patients (de Ataide et al., 2018). In addition, the kidney-tonifying botanical drug, *Cullen corylifolium* (L.) Medik. [Fabaceae], caused ALF as well (Li et al., 2019b). Moreover, pre-treatment of umbilical cord-derived mesenchymal stem cells (UC-MSCs) with asarinin significantly promotes the immunosuppressive effects of MSC after HSC transplantation (He et al., 2021), whereas it may have multiple cytotoxic effects, including arrhythmia, respiratory center depression, hepatotoxicity, and nephrotoxicity (Jeong et al., 2018). These are Chinese medicines commonly used in the clinical practice of MI; thus, attention must be paid when using these botanical drugs. First, it is recommended to take botanical drugs following the doctors’ instructions with a moderate dose. Second, processing (Paozhi) through steaming, boiling, stewing, refined honey, stir-frying, and calcining can directly reduce the contents of toxic constituents (Wu et al., 2018). Eventually, this not only alleviates the side effect of TCM but also improves oral absorption and bioavailability by using modern methods and materials to modify the TCM dosage form, such as lipid nanocarriers, polymeric nanocarriers, inorganic nanocarriers, and hybrid nanocarriers (Liu and Feng, 2015).

## Conclusion

Stem cell-based therapy after MI has made excellent progress in the last decade, whereas its drawbacks, such as low survival rate, low differentiation rate, and strong immunogenicity, severely limited the clinical application of this therapy. Our review showed that TCM has a great potential to compensate for the limitation of stem cells and can thus work together in preventing and treating MI. Based on TCM theories, we further summarized the mechanisms of Chinese medicine treatment in stem cell therapy by the commonly used prescriptions discussed previously. It seems that the role of TCM differs in different stages of stem cell therapy: 1) during the proliferation of stem cells, TCM mainly functions by tonifying the spleen and kidneys, nourishing qi and blood; 2) during the migration of stem cells, TCM mainly functions by tonifying the qi and activating blood; 3) during the differentiation of stem cells, TCM mainly functions by tonifying the kidneys and activating blood; and 4) when stem cells reach the infarct region, TCM can protect them even under hostile microenvironments by tonifying the qi and activating blood. In conclusion, tonifying the spleen and kidneys, replenishing qi, and activating blood are the basic therapeutic principles of TCM throughout the stem cell therapy. The principles allow us to choose the appropriate Chinese medicines in different stages of stem cell therapy for a more defined and precise functional study.

## Supplementary material

Supplementary Table S1 Composition of TCM prescription.

## Glossary

CVDs: cardiovascular diseases
MI: myocardial infarction
IHD: ischemic heart disease
ESCs: embryonic stem cells
ASCs: adult stem cells
TCM: traditional Chinese medicine
BMCs: bone marrow cells
HSCs: hematopoietic stem cells
MSCs: mesenchymal stem cells
RCSCs: resident cardiac stem cells
iPSCs: induced pluripotent stem cells
SMs: skeletal myoblasts
ADSCs: adipose-derived stem cells
AMI: acute myocardial infarction
LVEF: left ventricular ejection fraction
EPCs: endothelial progenitor cells
BMSCs: bone mesenchymal stem cells
ROS: reactive oxygen species
PCI: percutaneous coronary intervention
CM: conditional medium
SDE: Si–Wu decoction
EBM: epimedium brevicornum Maxim
ICA: icariin
PT: Plastrum testudinis
BMP4: bone morphogenetic protein 4
FP: Fructus Psoraleae
BYD: Buzhong Yiqi decoction
HA: hexadecanoic acid
SalB: salvianolic acid B
RGO: Rehmannia glutinosa oligosaccharide
APS: Angelica polysaccharides
SMSCs: skeletal muscle satellite cells
TCMI: TCM injection
DI: Danshen injection
TGF-β: transforming growth factor-β
TNF-α: tumor necrosis factor-α
SDF-1: stromal-derived factor-1
HGF: hepatocyte growth factor
GXDS: Guanxin Danshen
CHSIBA: Shen invigorating and blood activating
DHI: Danhong injection
SMB: Salvia miltiorrhiza Bunge
YWHR: Yiqi Wenyang Huoxue recipe
TIIA: Tanshinone IIA
PNS: Panax notoginseng saponins
RSV: resveratrol
MK: Musk ketonem
GJ: Geum japonicum
SLF: Shuanglong formula
VECs: vascular endothelial cells
XZD: Xuefu Zhuyu decoction
GBPYS: Gu Ben Pei Yuan San
CMECs: Cardiac microvascular endothelial cells
QLC: Qiliqiangxin capsule
XFZYD: Xuefu Zhuyu decoction
TXL: Tongxinluo
TPG: Total paeony glucosides
TMP: Tetramethylpyrazine
PPARγ: peroxisome proliferator-activated receptor γ
AS-IV: astragaloside IV
THSWD: Taohong Siwu decoction
NGR1: Notoginsenoside R1
RCT: random clinical trials
ALF: acute liver failure
UC-MSC: umbilical cord-derived mesenchymal stem cells

## References

[B1] Afjeh-Dana E. Naserzadeh P. Moradi E. Hosseini N. Seifalian A. M. Ashtari B.(2022). Stem cell differentiation into cardiomyocytes: Current methods and emerging approaches. Stem Cell. Rev. Rep. 18 (6). 10.1007/s12015-021-10280-1 35508757

[B2] Bacakova L. Zarubova J. Travnickova M. Musilkova J. Pajorova J. Slepicka P. (2018). Stem cells: Their source, potency and use in regenerative therapies with focus on adipose-derived stem cells - a review. Biotechnol. Adv. 36 (4), 1111–1126. 10.1016/j.biotechadv.2018.03.011 29563048

[B3] Beltrami A. P. Barlucchi L. Torella D. Baker M. Limana F. Chimenti S. (2003). Adult cardiac stem cells are multipotent and support myocardial regeneration. Cell. 114 (6), 763–776. 10.1016/s0092-8674(03)00687-1 14505575

[B4] Berger S. Aronson D. Lavie P. Lavie L. (2013). Endothelial progenitor cells in acute myocardial infarction and sleep-disordered breathing. Am. J. Respir. Crit. Care Med. 187 (1), 90–98. 10.1164/rccm.201206-1144OC 23155141

[B5] Bian X. Ma K. Zhang C. Fu X. (2019). Therapeutic angiogenesis using stem cell-derived extracellular vesicles: An emerging approach for treatment of ischemic diseases. Stem Cell. Res. Ther. 10 (1), 158. 10.1186/s13287-019-1276-z 31159859 PMC6545721

[B6] Blocki A. Beyer S. Dewavrin J. Y. Goralczyk A. Wang Y. Peh P. (2015). Microcapsules engineered to support mesenchymal stem cell (MSC) survival and proliferation enable long-term retention of MSCs in infarcted myocardium. Biomaterials 53, 12–24. 10.1016/j.biomaterials.2015.02.075 25890702

[B7] Bulati M. Miceli V. Gallo A. Amico G. Carcione C. Pampalone M. (2020). The immunomodulatory properties of the human amnion-derived mesenchymal stromal/stem cells are induced by INF-γ produced by activated lymphomonocytes and are mediated by cell-to-cell contact and soluble factors. Front. Immunol. 11, 54. 10.3389/fimmu.2020.00054 32117234 PMC7028706

[B8] Cao Y. Wang J. Su G. Wu Y. Bai R. Zhang Q. (2016). Anti-myocardial ischemia effect of Syringa pinnatifolia Hemsl. by inhibiting expression of cyclooxygenase-1 and -2 in myocardial tissues of mice. J. Ethnopharmacol. 187, 259–268. 10.1016/j.jep.2016.04.039 27130642

[B9] Carvalho E. Verma P. Hourigan K. Banerjee R. (2015). Myocardial infarction: Stem cell transplantation for cardiac regeneration. Regen. Med. 10 (8), 1025–1043. 10.2217/rme.15.63 26563414

[B10] Chamberlain S. J. (2016). Disease modelling using human iPSCs. Hum. Mol. Genet. 25 (R2), R173–r181. 10.1093/hmg/ddw209 27493026

[B11] Chen C. Dai P. Nan L. Lu R. Wang X. Tian Y. (2021). Isolation and characterization of endothelial progenitor cells from canine bone marrow. Biotech. Histochem. 96 (2), 85–93. 10.1080/10520295.2020.1762001 32476489

[B12] Chen D. F. Du S. H. Zhang H. L. Li H. Zhou J. H. Li Y. W. (2009). Autocrine BMP4 signaling involves effect of cholesterol myristate on proliferation of mesenchymal stem cells. Steroids 74 (13-14), 1066–1072. 10.1016/j.steroids.2009.08.008 19723531

[B13] Chen D. F. Li X. Xu Z. Liu X. Du S. H. Li H. (2010). Hexadecanoic acid from Buzhong Yiqi decoction induced proliferation of bone marrow mesenchymal stem cells. J. Med. Food 13 (4), 967–970. 10.1089/jmf.2009.1293 20482257

[B14] Chen D. F. Zeng H. P. Du S. H. Li H. Zhou J. H. Li Y. W. (2007). Extracts from Plastrum testudinis promote proliferation of rat bone-marrow-derived mesenchymal stem cells. Cell. Prolif. 40 (2), 196–212. 10.1111/j.1365-2184.2007.00431.x 17472727 PMC6496535

[B15] Chen J. Wei J. Huang Y. Ma Y. Ni J. Li M. (2018). Danhong injection enhances the therapeutic efficacy of mesenchymal stem cells in myocardial infarction by promoting angiogenesis. Front. Physiol. 9, 991. 10.3389/fphys.2018.00991 30093864 PMC6070728

[B16] Chen R. Chen W. Huang X. Rui Q. (2021). Tanshinone IIA attenuates heart failure via inhibiting oxidative stress in myocardial infarction rats. Mol. Med. Rep. 23 (6), 404. 10.3892/mmr.2021.12043 33786621 PMC8025468

[B17] Chen S. L. Fang W. W. Qian J. Ye F. Liu Y. h. Shan S. j. (2004). Improvement of cardiac function after transplantation of autologous bone marrow mesenchymal stem cells in patients with acute myocardial infarction. Chin. Med. J. 117 (10), 1443–1448. 15498362

[B18] Cheng L. Chen H. Yao X. Qi G. Liu H. Lee K. (2009). A plant-derived remedy for repair of infarcted heart. PLoS One 4 (2), e4461. 10.1371/journal.pone.0004461 19221596 PMC2637970

[B19] Cheng S. Zhang X. Feng Q. Chen J. Shen L. Yu P. (2019). Astragaloside IV exerts angiogenesis and cardioprotection after myocardial infarction via regulating PTEN/PI3K/Akt signaling pathway. Life Sci. 227, 82–93. 10.1016/j.lfs.2019.04.040 31004658

[B20] Cui B. Zheng Y. Zhou X. Zhu J. Zhuang J. Liang Q. (2019). Repair of adult mammalian heart after damages by oral intake of Gu ben Pei yuan san. Front. Physiol. 10, 607. 10.3389/fphys.2019.00607 31191336 PMC6541202

[B21] Davy P. M. Lye K. D. Mathews J. Owens J. B. Chow A. Y. Wong L. (2015). Human adipose stem cell and ASC-derived cardiac progenitor cellular therapy improves outcomes in a murine model of myocardial infarction. Stem Cells Cloning 8, 135–148. 10.2147/SCCAA.S86925 26604802 PMC4631407

[B22] de Ataide E. C. Reges Perales S. de Oliveira Peres M. A. Bastos Eloy da Costa L. QuarellaF. Valerini F. G. (2018). Acute liver failure induced by Carthamus tinctorius oil: Case reports and literature review. Transpl. Proc. 50 (2), 476–477. 10.1016/j.transproceed.2018.01.010 29579831

[B23] Deng S. Zhou X. Ge Z. Song Y. Wang H. Liu X. (2019). Exosomes from adipose-derived mesenchymal stem cells ameliorate cardiac damage after myocardial infarction by activating S1P/SK1/S1PR1 signaling and promoting macrophage M2 polarization. Int. J. Biochem. Cell. Biol. 114, 105564. 10.1016/j.biocel.2019.105564 31276786

[B24] Dib N. Dinsmore J. Lababidi Z. White B. Moravec S. Campbell A. (2009). One-year follow-up of feasibility and safety of the first U.S., randomized, controlled study using 3-dimensional guided catheter-based delivery of autologous skeletal myoblasts for ischemic cardiomyopathy (CAuSMIC study). JACC. Cardiovasc. Interv. 2 (1), 9–16. 10.1016/j.jcin.2008.11.003 19463392

[B25] Ding R. Jiang X. Ha Y. Wang Z. Guo J. Jiang H. (2015). Activation of Notch1 signalling promotes multi-lineage differentiation of c-kit(POS)/NKX2.5(POS) bone marrow stem cells: Implication in stem cell translational medicine. Stem Cell. Res. Ther. 6 (1), 91. 10.1186/s13287-015-0085-2 25956503 PMC4446115

[B26] Drowley L. Koonce C. Peel S. Jonebring A. Plowright A. T. Kattman S. J. (2016). Human induced pluripotent stem cell-derived cardiac progenitor cells in phenotypic screening: A transforming growth factor-β type 1 receptor kinase inhibitor induces efficient cardiac differentiation. Stem Cells Transl. Med. 5 (2), 164–174. 10.5966/sctm.2015-0114 26683871 PMC4729552

[B27] Eldaly A. S. Mashaly S. M. Fouda E. Emam O. S. Aglan A. Abuasbeh J. (2022). Systemic anti-inflammatory effects of mesenchymal stem cells in burn: A systematic review of animal studies. J. Clin. Transl. Res. 8 (4), 276–291. 35991083 PMC9389574

[B28] Eschenhagen T. Bolli R. Braun T. Field L. J. Fleischmann B. K. Frisen J. (2017). Cardiomyocyte regeneration: A consensus statement. Circulation 136 (7), 680–686. 10.1161/CIRCULATIONAHA.117.029343 28684531 PMC5557671

[B29] Faiella W. Atoui R. (2016). Therapeutic use of stem cells for cardiovascular disease. Clin. Transl. Med. 5 (1), 34. 10.1186/s40169-016-0116-3 27539581 PMC4990528

[B30] Fan X. Li X. Lv S. Wang Y. Zhao Y. Luo G. (2010). Comparative proteomics research on rat MSCs differentiation induced by Shuanglong Formula. J. Ethnopharmacol. 131 (3), 575–580. 10.1016/j.jep.2010.07.036 20659544

[B31] Fisher S. A. Brunskill S. J. Doree C. Taggart D. P. Martin-Rendon E. (2014). Stem cell therapy for chronic ischaemic heart disease and congestive heart failure. Cochrane Database Syst. Rev. 12 (4), Cd007888. 10.1002/14651858.CD007888.pub3 PMC6463978 28012165

[B32] Fisher S. A. Doree C. Mathur A. Taggart D. P. Martin-Rendon E. (2018). Cochrane corner: Stem cell therapy for chronic ischaemic heart disease and congestive heart failure. Heart 104 (1), 8–10. 10.1136/heartjnl-2017-311684 28607164

[B33] Franchi F. Ramaswamy V. Olthoff M. Peterson K. M. Paulmurugan R. Rodriguez-Porcel M. (2020). The myocardial microenvironment modulates the biology of transplanted mesenchymal stem cells. Mol. Imaging Biol. 22 (4), 948–957. 10.1007/s11307-019-01470-y 31907845 PMC7335681

[B34] Fu X. He Q. Tao Y. Wang M. Wang W. Wang Y. (2021). Recent advances in tissue stem cells. Sci. China. Life Sci. 64 (12), 1998–2029. 10.1007/s11427-021-2007-8 34865207

[B35] Fu Y. Huang C. Xu X. Gu H. Ye Y. Jiang C. (2015). Direct reprogramming of mouse fibroblasts into cardiomyocytes with chemical cocktails. Cell. Res. 25 (9), 1013–1024. 10.1038/cr.2015.99 26292833 PMC4559819

[B36] Gao D. Wu L. Y. Jiao Y. H. Chen W. y. Chen Y. Kaptchuk T. J. (2010). The effect of Xuefu Zhuyu Decoction on *in vitro* endothelial progenitor cell tube formation. Chin. J. Integr. Med. 16 (1), 50–53. 10.1007/s11655-010-0050-y 20131036 PMC2819674

[B37] Gao H. Bo Z. Wang Q. Luo L. Zhu H. Ren Y. (2019). Salvanic acid B inhibits myocardial fibrosis through regulating TGF-β1/Smad signaling pathway. Biomed. Pharmacother. 110, 685–691. 10.1016/j.biopha.2018.11.098 30553195

[B38] Gao L. R. Pei X. T. Ding Q. A. Chen Y. Zhang N. K. Chen H. Y. (2013). A critical challenge: Dosage-related efficacy and acute complication intracoronary injection of autologous bone marrow mesenchymal stem cells in acute myocardial infarction. Int. J. Cardiol. 168 (4), 3191–3199. 10.1016/j.ijcard.2013.04.112 23651816

[B39] Gao X. R. Xu H. J. Wang L. F. Liu C. B. Yu F. (2017). Mesenchymal stem cell transplantation carried in SVVYGLR modified self-assembling peptide promoted cardiac repair and angiogenesis after myocardial infarction. Biochem. Biophys. Res. Commun. 491 (1), 112–118. 10.1016/j.bbrc.2017.07.056 28709866

[B40] Grajek S. Popiel M. Gil L. Breborowicz P. Lesiak M. Czepczynski R. (2010). Influence of bone marrow stem cells on left ventricle perfusion and ejection fraction in patients with acute myocardial infarction of anterior wall: Randomized clinical trial: Impact of bone marrow stem cell intracoronary infusion on improvement of microcirculation. Eur. Heart J. 31 (6), 691–702. 10.1093/eurheartj/ehp536 20022872

[B41] Gu F. Jiang J. Wang S. Feng T. Zhou Y. Ma Y. (2019). An experimental research into the potential therapeutic effects of Anti-Osteoporosis Decoction and Yougui Pill on ovariectomy-induced osteoporosis. Am. J. Transl. Res. 11 (9), 6032–6039. 31632571 PMC6789260

[B42] Gu M. Mordwinkin N. M. Kooreman N. G. Lee J. Wu H. Hu S. (2015). Pravastatin reverses obesity-induced dysfunction of induced pluripotent stem cell-derived endothelial cells via a nitric oxide-dependent mechanism. Eur. Heart J. 36 (13), 806–816. 10.1093/eurheartj/ehu411 25368203 PMC4381134

[B43] Guan J. Wang G. Wang J. Zhang Z. Fu Y. Cheng L. (2022). Chemical reprogramming of human somatic cells to pluripotent stem cells. Nature 605 (7909), 325–331. 10.1038/s41586-022-04593-5 35418683

[B44] Guo H. D. Cui G. H. Tian J. X. Lu P. P. Zhu Q. C. Lv R. (2014). Transplantation of salvianolic acid B pretreated mesenchymal stem cells improves cardiac function in rats with myocardial infarction through angiogenesis and paracrine mechanisms. Int. J. Cardiol. 177 (2), 538–542. 10.1016/j.ijcard.2014.08.104 25189503

[B45] Guo Y. Yu Y. Hu S. Chen Y. Shen Z. (2020). The therapeutic potential of mesenchymal stem cells for cardiovascular diseases. Cell. Death Dis. 11 (5), 349. 10.1038/s41419-020-2542-9 32393744 PMC7214402

[B46] Gurusamy N. Alsayari A. Rajasingh S. Rajasingh J. (2018). Adult stem cells for regenerative therapy. Prog. Mol. Biol. Transl. Sci. 160, 1–22. 10.1016/bs.pmbts.2018.07.009 30470288

[B47] Haider K. H. (2018). Bone marrow cell therapy and cardiac reparability: Better cell characterization will enhance clinical success. Regen. Med. 13 (4), 457–475. 10.2217/rme-2017-0134 29985118

[B48] Han X. J. Li H. Liu C. B. Luo Z. R. Wang Q. L. Mou F. F. (2019). Guanxin Danshen Formulation improved the effect of mesenchymal stem cells transplantation for the treatment of myocardial infarction probably via enhancing the engraftment. Life Sci. 233, 116740. 10.1016/j.lfs.2019.116740 31398416

[B49] He H. Yang T. Li F. Zhang L. Ling X. (2021). A novel study on the immunomodulatory effect of umbilical cord derived mesenchymal stem cells pretreated with traditional Chinese medicine Asarinin. Int. Immunopharmacol. 100, 108054. 10.1016/j.intimp.2021.108054 34492537

[B50] Hedderich J. El Bagdadi K. Angele P. Grassel S. Meurer A. Straub R. H. (2020). Norepinephrine inhibits the proliferation of human bone marrow-derived mesenchymal stem cells via β2-adrenoceptor-mediated ERK1/2 and PKA phosphorylation. Int. J. Mol. Sci. 21 (11), E3924. 10.3390/ijms21113924 32486305 PMC7312191

[B51] Heinrich M. Appendino G. Efferth T. Furst R. Izzo A. A. Kayser O. (2020). Best practice in research - overcoming common challenges in phytopharmacological research. J. Ethnopharmacol. 246, 112230. 10.1016/j.jep.2019.112230 31526860

[B52] Henry T. D. Tomey M. I. Tamis-Holland J. E. Thiele H. Rao S. V. Menon V. (2021). Invasive management of acute myocardial infarction complicated by cardiogenic shock: A scientific statement from the American heart association. Circulation 143 (15), e815–e829. 10.1161/CIR.0000000000000959 33657830

[B53] Herreros J. Prósper F. Perez A. Gavira J. J. Garcia-Velloso M. J. Barba J. (2003). Autologous intramyocardial injection of cultured skeletal muscle-derived stem cells in patients with non-acute myocardial infarction. Eur. Heart J. 24 (22), 2012–2020. 10.1016/j.ehj.2003.09.012 14613737

[B54] Hong W. Tatsuo S. Shou-Dong W. Qian Z. Jian-Feng H. Jue W. (2015). Resveratrol upregulates cardiac SDF-1 in mice with acute myocardial infarction through the deacetylation of cardiac p53. PLoS One 10 (6), e0128978. 10.1371/journal.pone.0128978 26053177 PMC4459949

[B55] Huang H. Xu Z. Qi Y. Zhang W. Zhang C. Jiang M. (2020). Exosomes from SIRT1-overexpressing ADSCs restore cardiac function by improving angiogenic function of EPCs. Mol. Ther. Nucleic Acids 21, 737–750. 10.1016/j.omtn.2020.07.007 32771925 PMC7412761

[B56] Hynes K. Menichanin D. Bright R. IvanovSki S. Hutmacher D. W. GronthoS S. (2015). Induced pluripotent stem cells: A new frontier for stem cells in dentistry. J. Dent. Res. 94 (11), 1508–1515. 10.1177/0022034515599769 26285811

[B57] Imanishi Y. Miyagawa S. Saito A. Kitagawa-Sakakida S. Sawa Y. (2011). Allogenic skeletal myoblast transplantation in acute myocardial infarction model rats. Transplantation 91 (4), 425–431. 10.1097/TP.0b013e3182052bca 21200367

[B58] Jeong M. Kim H. M. Lee J. S. Choi J. H. Jang D. S. (2018). (-)-Asarinin from the roots of asarum sieboldii induces apoptotic cell death via caspase activation in human ovarian cancer cells. Molecules 23 (8), E1849. 10.3390/molecules23081849 30044423 PMC6222791

[B59] Jia Q. Zhu R. Tian Y. Chen B. Li R. Li L. (2019). Salvia miltiorrhiza in diabetes: A review of its pharmacology, phytochemistry, and safety. Phytomedicine 58, 152871. 10.1016/j.phymed.2019.152871 30851580

[B60] Khan M. Nickoloff E. Abramova T. Johnson J. Verma S. K. Krishnamurthy P. (2015). Embryonic stem cell-derived exosomes promote endogenous repair mechanisms and enhance cardiac function following myocardial infarction. Circ. Res. 117 (1), 52–64. 10.1161/CIRCRESAHA.117.305990 25904597 PMC4482130

[B61] Kim S. W. Kim H. W. Huang W. Okada M. Welge J. A. Wang Y. (2013). Cardiac stem cells with electrical stimulation improve ischaemic heart function through regulation of connective tissue growth factor and miR-378. Cardiovasc. Res. 100 (2), 241–251. 10.1093/cvr/cvt192 24067999 PMC3797629

[B62] Lagarkova M. A. (2019). Such various stem cells. Biochemistry. 84 (3), 187–189. 10.1134/S0006297919030015 31221057

[B63] Laplane L. Solary E. (2019). Towards a classification of stem cells. Elife 8, e46563. 10.7554/eLife.46563 30864951 PMC6415933

[B64] Lee T. L. Lai T. C. Lin S. R. Chen Y. C. Pu C. M. Lee I. T. (2021). Conditioned medium from adipose-derived stem cells attenuates ischemia/reperfusion-induced cardiac injury through the microRNA-221/222/PUMA/ETS-1 pathway. Theranostics 11 (7), 3131–3149. 10.7150/thno.52677 33537078 PMC7847683

[B65] Li A. Gao M. Zhao N. Li P. Zhu J. Li W. (2019). Acute liver failure associated with fructus Psoraleae: A case report and literature review. BMC Complement. Altern. Med. 19 (1), 84. 10.1186/s12906-019-2493-9 30975110 PMC6458792

[B66] Li H. Zhu J. Xu Y. W. Mou F. F. Shan X. L. Wang Q. L. (2022). Notoginsenoside R1-loaded mesoporous silica nanoparticles targeting the site of injury through inflammatory cells improves heart repair after myocardial infarction. Redox Biol. 54, 102384. 10.1016/j.redox.2022.102384 35777198 PMC9287735

[B67] Li L. Chen X. Wang W. E. Zeng C., (2016). How to improve the survival of transplanted mesenchymal stem cell in ischemic heart? Stem Cells Int. 2016, 9682757. 10.1155/2016/9682757 26681958 PMC4670674

[B68] Li M. Yu C. M. Cheng L. Wang M. Gu X. Lee K. H. (2006). Repair of infarcted myocardium by an extract of Geum japonicum with dual effects on angiogenesis and myogenesis. Clin. Chem. 52 (8), 1460–1468. 10.1373/clinchem.2006.068247 16873297

[B69] Li N. Yang Y. J. Cui H. H. Zhang Q. Jin C. Qian H. Y. (2014). Tongxinluo decreases apoptosis of mesenchymal stem cells concentration-dependently under hypoxia and serum deprivation conditions through the AMPK/eNOS pathway. J. Cardiovasc. Pharmacol. 63 (3), 265–273. 10.1097/FJC.0000000000000044 24220313

[B70] Li X. Zhao H. Qi C. Zeng Y. Xu F. Du Y. (2015). Direct intercellular communications dominate the interaction between adipose-derived MSCs and myofibroblasts against cardiac fibrosis. Protein Cell. 6 (10), 735–745. 10.1007/s13238-015-0196-7 26271509 PMC4598323

[B71] Li Z. Solomonidis E. G. Meloni M. Taylor R. S. Duffin R. Dobie R. (2019). Single-cell transcriptome analyses reveal novel targets modulating cardiac neovascularization by resident endothelial cells following myocardial infarction. Eur. Heart J. 40 (30), 2507–2520. 10.1093/eurheartj/ehz305 31162546 PMC6685329

[B72] Lin M. Liu X. Zheng H. Huang X. Wu Y. Huang A. (2020). IGF-1 enhances BMSC viability, migration, and anti-apoptosis in myocardial infarction via secreted frizzled-related protein 2 pathway. Stem Cell. Res. Ther. 11 (1), 22. 10.1186/s13287-019-1544-y 31918758 PMC6953226

[B73] Lin X. Peng P. Cheng L. Chen S. Li K. Li Z. Y. (2012). A natural compound induced cardiogenic differentiation of endogenous MSCs for repair of infarcted heart. Differentiation. 83 (1), 1–9. 10.1016/j.diff.2011.09.001 22099171

[B74] Liu J. Jiang M. Deng S. Lu J. Huang H. Zhang Y. (2018). miR-93-5p-Containing exosomes treatment attenuates acute myocardial infarction-induced myocardial damage. Mol. Ther. Nucleic Acids 11, 103–115. 10.1016/j.omtn.2018.01.010 29858047 PMC5852413

[B75] Liu Y. Feng N. (2015). Nanocarriers for the delivery of active ingredients and fractions extracted from natural products used in traditional Chinese medicine (TCM). Adv. Colloid Interface Sci. 221, 60–76. 10.1016/j.cis.2015.04.006 25999266

[B76] Liu Y. Hao F. Zhang H. Cao D. Lu X. Li X. (2013). Panax notoginseng saponins promote endothelial progenitor cell mobilization and attenuate atherosclerotic lesions in apolipoprotein E knockout mice. Cell. Physiol. biochem. 32 (4), 814–826. 10.1159/000354484 24080960

[B77] Lo B. Parham L. (2009). Ethical issues in stem cell research. Endocr. Rev. 30 (3), 204–213. 10.1210/er.2008-0031 19366754 PMC2726839

[B78] Luo C. Wang H. Chen X. Cui Y. Li H. Long J. (2013). Protection of H9c2 rat cardiomyoblasts against oxidative insults by total paeony glucosides from Radix Paeoniae Rubrae. Phytomedicine 21 (1), 20–24. 10.1016/j.phymed.2013.08.002 24035226

[B79] Luo Z. R. Li H. Xiao Z. X. Shao S. J. Zhao T. T. Zhao Y. (2019). Taohong siwu decoction exerts a beneficial effect on cardiac function by possibly improving the microenvironment and decreasing mitochondrial fission after myocardial infarction. Cardiol. Res. Pract. 2019, 5198278. 10.1155/2019/5198278 31885903 PMC6925791

[B80] Ma R. Liang J. Huang W. Guo L. Cai W. Wang L. (2018). Electrical stimulation enhances cardiac differentiation of human induced pluripotent stem cells for myocardial infarction therapy. Antioxid. Redox Signal. 28 (5), 371–384. 10.1089/ars.2016.6766 27903111 PMC5770128

[B81] Madigan M. Atoui R. (2018). Therapeutic use of stem cells for myocardial infarction. Bioeng. (Basel) 5 (2), E28. 10.3390/bioengineering5020028 PMC6027340 29642402

[B82] Makkar R. R. Smith R. R. Cheng K. Malliaras K. Thomson L. E. Berman D. (2012). Intracoronary cardiosphere-derived cells for heart regeneration after myocardial infarction (CADUCEUS): A prospective, randomised phase 1 trial. Lancet 379 (9819), 895–904. 10.1016/S0140-6736(12)60195-0 22336189 PMC4326004

[B83] Malchenko S. Xie J. de Fatima Bonaldo M. Vanin E. F. Bhattacharyya B. J. Belmadani A. (2014). Onset of rosette formation during spontaneous neural differentiation of hESC and hiPSC colonies. Gene 534 (2), 400–407. 10.1016/j.gene.2013.07.101 23954875

[B84] Menasché P. Alfieri O. Janssens S. McKenna W. Reichenspurner H. Trinquart L. (2008). The myoblast autologous grafting in ischemic cardiomyopathy (MAGIC) trial: First randomized placebo-controlled study of myoblast transplantation. Circulation 117 (9), 1189–1200. 10.1161/CIRCULATIONAHA.107.734103 18285565

[B85] Miao C. Lei M. Hu W. Han S. Wang Q. (2017). A brief review: The therapeutic potential of bone marrow mesenchymal stem cells in myocardial infarction. Stem Cell. Res. Ther. 8 (1), 242. 10.1186/s13287-017-0697-9 29096705 PMC5667518

[B86] Müller P. Lemcke H. David R. (2018). Stem cell therapy in heart diseases - cell types, mechanisms and improvement strategies. Cell. Physiol. biochem. 48 (6), 2607–2655. 10.1159/000492704 30121644

[B87] Nasseri B. A. Ebell W. Dandel M. Kukucka M. Gebker R. Doltra A. (2014). Autologous CD133+ bone marrow cells and bypass grafting for regeneration of ischaemic myocardium: The Cardio133 trial. Eur. Heart J. 35 (19), 1263–1274. 10.1093/eurheartj/ehu007 24497345

[B88] Penn M. S. Mangi A. A. (2008). Genetic enhancement of stem cell engraftment, survival, and efficacy. Circ. Res. 102 (12), 1471–1482. 10.1161/CIRCRESAHA.108.175174 18566313 PMC2668244

[B89] Ponsuksili S. Murani E. Hadlich F. Perdomo-Sabogal A. Trakooljul N. Oster M. (2022). Genetic regulation and variation of expression of miRNA and mRNA transcripts in fetal muscle tissue in the context of sex, dam and variable fetal weight. Biol. Sex. Differ. 13 (1), 24. 10.1186/s13293-022-00433-3 35550009 PMC9103043

[B90] Qi K. Li L. Li X. Zhao J. Wang Y. You S. (2015). Cardiac microvascular barrier function mediates the protection of Tongxinluo against myocardial ischemia/reperfusion injury. PLoS One 10 (3), e0119846. 10.1371/journal.pone.0119846 25781461 PMC4363146

[B91] Qian H. Y. Yang Y. J. Huang J. Gao R. l. Dou K. f. Yang G. s. (2007). Effects of Tongxinluo-facilitated cellular cardiomyoplasty with autologous bone marrow-mesenchymal stem cells on postinfarct swine hearts. Chin. Med. J. 120 (16), 1416–1425. 10.1097/00029330-200708020-00008 17825171

[B92] Qin S. Zhou W. Liu S. Chen P. Wu H. (2015). Icariin stimulates the proliferation of rat bone mesenchymal stem cells via ERK and p38 MAPK signaling. Int. J. Clin. Exp. Med. 8 (5), 7125–7133. 26221250 PMC4509195

[B93] Rigaud V. O. C. Hoy R. Mohsin S. Khan M. (2020). Stem cell metabolism: Powering cell-based therapeutics. Cells 9 (11), E2490. 10.3390/cells9112490 33207756 PMC7696341

[B94] Roth G. A. Mensah G. A. Johnson C. O. Addolorato G. Ammirati E. Baddour L. M. (2020). Global burden of cardiovascular diseases and risk factors, 1990-2019: Update from the GBD 2019 study. J. Am. Coll. Cardiol. 76 (25), 2982–3021. 10.1016/j.jacc.2020.11.010 33309175 PMC7755038

[B95] Samak M. Hinkel R. (2019). Stem cells in cardiovascular medicine: Historical overview and future prospects. Cells 8 (12), E1530. 10.3390/cells8121530 31783680 PMC6952821

[B96] Shafei A. E. Ali M. A. Ghanem H. G. Shehata A. I. Abdelgawad A. A. Handal H. R. (2018). Mechanistic effects of mesenchymal and hematopoietic stem cells: New therapeutic targets in myocardial infarction. J. Cell. Biochem. 119 (7), 5274–5286. 10.1002/jcb.26637 29266431

[B97] Shen X. Pan B. Zhou H. Liu L. Lv T. Zhu J. (2017). Differentiation of mesenchymal stem cells into cardiomyocytes is regulated by miRNA-1-2 via WNT signaling pathway. J. Biomed. Sci. 24 (1), 29. 10.1186/s12929-017-0337-9 28490365 PMC5424345

[B98] Shi W. L. Zhao J. Yuan R. Lu Y. Xin Q. Q. Liu Y. (2019). Combination of Ligusticum chuanxiong and radix Paeonia promotes angiogenesis in ischemic myocardium through Notch signalling and mobilization of stem cells. Evid. Based. Complement. Altern. Med. 2019, 7912402. 10.1155/2019/7912402 PMC6398078 30906416

[B99] Siminiak T. Kalawski R. Fiszer D. Jerzykowska O. Rzezniczak J. Rozwadowska N. (2004). Autologous skeletal myoblast transplantation for the treatment of postinfarction myocardial injury: phase I clinical study with 12 months of follow-up. Am. Heart J. 148 (3), 531–537. 10.1016/j.ahj.2004.03.043 15389244

[B100] Skuk D. Tremblay J. P. (2019). Myotubes formed de novo by myoblasts injected into the scar of myocardial infarction persisted for 16 Years in a patient: Importance for regenerative medicine in degenerative myopathies. Stem Cells Transl. Med. 8 (3), 313–314. 10.1002/sctm.18-0202 30506986 PMC6392342

[B101] Sun Y. Zhang J. Qian N. Sima G. Zhang J. Zhong J. (2018). Comparison of the osteogenic differentiation of orofacial bone marrow stromal cells prior to and following marsupialization in patients with odontogenic cyst. Mol. Med. Rep. 17 (1), 988–994. 10.3892/mmr.2017.7949 29115541 PMC5780180

[B102] Tendera M. Wojakowski W. Ruzyłło W. Chojnowska L. Kepka C. Tracz W. (2009). Intracoronary infusion of bone marrow-derived selected CD34+CXCR4+ cells and non-selected mononuclear cells in patients with acute STEMI and reduced left ventricular ejection fraction: Results of randomized, multicentre myocardial regeneration by intracoronary infusion of selected population of stem cells in acute myocardial infarction (REGENT) trial. Eur. Heart J. 30 (11), 1313–1321. 10.1093/eurheartj/ehp073 19208649

[B103] Terajima Y. Shimizu T. Tsuruyama S. Sekine H. Ishii H. Yamazaki K. (2014). Autologous skeletal myoblast sheet therapy for porcine myocardial infarction without increasing risk of arrhythmia. Cell. Med. 6 (3), 99–109. 10.3727/215517913X672254 26858886 PMC4733831

[B104] Tsao C. W. Aday A. W. Almarzooq Z. I. Alonso A. Beaton A. Z. Bittencourt M. S. (2022). Heart disease and stroke statistics-2022 update: A report from the American heart association. Circulation 145 (8), e153–e639. 10.1161/CIR.0000000000001052 35078371

[B105] Tzahor E. Poss K. D. (2017). Cardiac regeneration strategies: Staying young at heart. Science 356 (6342), 1035–1039. 10.1126/science.aam5894 28596337 PMC5614484

[B106] Ulus A. T. Mungan C. Kurtoglu M. Celikkan F. T. Akyol M. Sucu M. (2020). Intramyocardial transplantation of umbilical cord mesenchymal stromal cells in chronic ischemic cardiomyopathy: A controlled, randomized clinical trial (HUC-heart trial). Int. J. Stem Cells 13 (3), 364–376. 10.15283/ijsc20075 32840230 PMC7691850

[B107] Uygur A. Lee R. T. (2016). Mechanisms of cardiac regeneration. Dev. Cell. 36 (4), 362–374. 10.1016/j.devcel.2016.01.018 26906733 PMC4768311

[B108] Vagnozzi R. J. Maillet M. Sargent M. A. Khalil H. Johansen A. K. Z. Schwanekamp J. A. (2020). An acute immune response underlies the benefit of cardiac stem cell therapy. Nature 577 (7790), 405–409. 10.1038/s41586-019-1802-2 31775156 PMC6962570

[B109] Valiente-Alandi I. Albo-Castellanos C. Herrero D. Sanchez I. Bernad A. (2016). Bmi1 (+) cardiac progenitor cells contribute to myocardial repair following acute injury. Stem Cell. Res. Ther. 7 (1), 100. 10.1186/s13287-016-0355-7 27472922 PMC4967328

[B110] van der Spoel T. I. Jansen of Lorkeers S. J. Agostoni P. van Belle E. Gyongyosi M. Sluijter J. P. G. (2011). Human relevance of pre-clinical studies in stem cell therapy: Systematic review and meta-analysis of large animal models of ischaemic heart disease. Cardiovasc. Res. 91 (4), 649–658. 10.1093/cvr/cvr113 21498423

[B111] Wang C. Hou J. Du H. Yan S. Yang J. Wang Y. (2019). Anti-depressive effect of Shuangxinfang on rats with acute myocardial infarction: Promoting bone marrow mesenchymal stem cells mobilization and alleviating inflammatory response. Biomed. Pharmacother. 111, 19–30. 10.1016/j.biopha.2018.11.113 30553131

[B112] Wang J. N. Kan C. D. Lee L. T. Huang L. L. H. Hsiao Y. L. Chang A. H. (2021). Herbal extract from Codonopsis pilosula (franch.) Nannf. Enhances cardiogenic differentiation and improves the function of infarcted rat hearts. Life (Basel) 11 (5), 422. 10.3390/life11050422 34063127 PMC8148170

[B113] Wang M. Liu J. Zhou B. Xu R. Tao L. Ji M. (2012). Acute and sub-chronic toxicity studies of Danshen injection in Sprague-Dawley rats. J. Ethnopharmacol. 141 (1), 96–103. 10.1016/j.jep.2012.02.005 22343168

[B114] Wang T. T. Chen W. Zeng H. P. Chen D. F. (2012). Chemical components in extracts from Plastrum testudinis with proliferation-promoting effects on rat mesenchymal stem cells. Chem. Biol. Drug Des. 79 (6), 1049–1055. 10.1111/j.1747-0285.2012.01361.x 22339978

[B115] Wang X. Zhen L. Miao H. Sun Q. Yang Y. Que B. (2015). Concomitant retrograde coronary venous infusion of basic fibroblast growth factor enhances engraftment and differentiation of bone marrow mesenchymal stem cells for cardiac repair after myocardial infarction. Theranostics 5 (9), 995–1006. 10.7150/thno.11607 26155315 PMC4493537

[B116] Wei R. Yang J. Gao M. Wang H. Hou W. Mu Y. (2016). Infarcted cardiac microenvironment may hinder cardiac lineage differentiation of human embryonic stem cells. Cell. Biol. Int. 40 (11), 1235–1246. 10.1002/cbin.10679 27600481

[B117] Windmueller R. Leach J. P. Babu A. Zhou S. Morley M. P. Wakabayashi A. (2020). Direct comparison of mononucleated and binucleated cardiomyocytes reveals molecular mechanisms underlying distinct proliferative competencies. Cell. Rep. 30 (9), 3105–3116. e4. 10.1016/j.celrep.2020.02.034 32130910 PMC7194103

[B118] Wollert K. C. Meyer G. P. Müller-Ehmsen J. Tschope C. Bonarjee V. Larsen A. I. (2017). Intracoronary autologous bone marrow cell transfer after myocardial infarction: The BOOST-2 randomised placebo-controlled clinical trial. Eur. Heart J. 38 (39), 2936–2943. 10.1093/eurheartj/ehx188 28431003

[B119] Wu X. Reboll M. R. Korf-Klingebiel M. Wollert K. C. (2021). Angiogenesis after acute myocardial infarction. Cardiovasc. Res. 117 (5), 1257–1273. 10.1093/cvr/cvaa287 33063086

[B120] Wu X. Wang S. Lu J. Jing Y. Li M. Cao J. (2018). Seeing the unseen of Chinese herbal medicine processing (paozhi): Advances in new perspectives. Chin. Med. 13, 4. 10.1186/s13020-018-0163-3 29375653 PMC5773022

[B121] Xie J. Wang H. Song T. Wang Z. Li F. Ma J. (2013). Tanshinone IIA and astragaloside IV promote the migration of mesenchymal stem cells by up-regulation of CXCR4. Protoplasma 250 (2), 521–530. 10.1007/s00709-012-0435-1 22872094

[B122] Xiong Y. Tang R. Xu J. Jiang W. Gong Z. Zhang L. (2022). Sequential transplantation of exosomes and mesenchymal stem cells pretreated with a combination of hypoxia and Tongxinluo efficiently facilitates cardiac repair. Stem Cell. Res. Ther. 13 (1), 63. 10.1186/s13287-022-02736-z 35130979 PMC8822662

[B123] Xiong Y. Tang R. Xu J. Jiang W. Gong Z. Zhang L. (2022). Tongxinluo-pretreated mesenchymal stem cells facilitate cardiac repair via exosomal transfer of miR-146a-5p targeting IRAK1/NF-κB p65 pathway. Stem Cell. Res. Ther. 13 (1), 289. 10.1186/s13287-022-02969-y 35799283 PMC9264662

[B124] Xu H. Zhang Y. Wang P. Zhang J. Chen H. Zhang L. (2021). A comprehensive review of integrative pharmacology-based investigation: A paradigm shift in traditional Chinese medicine. Acta Pharm. Sin. B 11 (6), 1379–1399. 10.1016/j.apsb.2021.03.024 34221858 PMC8245857

[B125] Yan R. Yang Y. Chen Y. (2018). Pharmacokinetics of Chinese medicines: Strategies and perspectives. Chin. Med. 13, 24. 10.1186/s13020-018-0183-z 29743935 PMC5930430

[B126] Yu L. Tu Q. Han Q. Zhang L. Sui L. Zheng L. (2015). Adiponectin regulates bone marrow mesenchymal stem cell niche through a unique signal transduction pathway: An approach for treating bone disease in diabetes. Stem Cells 33 (1), 240–252. 10.1002/stem.1844 25187480 PMC4681406

[B127] Yu Y. Sun J. Liu J. Wang P. Wang C. (2020). Ginsenoside Re preserves cardiac function and ameliorates left ventricular remodeling in a rat model of myocardial infarction. J. Cardiovasc. Pharmacol. 75 (1), 91–97. 10.1097/FJC.0000000000000752 31599782

[B128] Zeng H. P. Wang T. T. Chen W. Wang C. Y. Chen D. F. Shen J. G. (2008). Characterization of chemical components in extracts from Si-Wu decoction with proliferation-promoting effects on rat mesenchymal stem cells. Bioorg. Med. Chem. 16 (9), 5109–5114. 10.1016/j.bmc.2008.03.024 18374577

[B129] Zhang J. S. He Q. Y. Huang T. Zhang B. X. (2011). Effects of panax notoginseng saponins on homing of C-kit+ bone mesenchymal stem cells to the infarction heart in rats. J. Tradit. Chin. Med. 31 (3), 203–208. 10.1016/s0254-6272(11)60043-5 21977864

[B130] Zhang J. Zhang B. Zhu H. Zhang Y. (2016). Insight into stem cells, microenvironment and methods for promoting blood circulation and removing stasis. Chin. J. Tissue Eng. Res. 20 (23), 3484–3490. 10.3969/j.issn.2095-4344.2016.23.021

[B131] Zhang J. Zhang B. (2018). Research on identity between kidney essence and stem cells. Chin. Archives Traditional Chin. Med. 36 (2), 326–328. 10.13193/j.issn.1673-7717.2018.02.017

[B132] Zhang R. Yu J. Zhang N. Li W. Wang J. Cai G. (2021). Bone marrow mesenchymal stem cells transfer in patients with ST-segment elevation myocardial infarction: Single-blind, multicenter, randomized controlled trial. Stem Cell. Res. Ther. 12 (1), 33. 10.1186/s13287-020-02096-6 33413636 PMC7791674

[B133] Zhang X. Y. Sun Y. Yang X. Y. Hu J. Y. Zheng R. Chen S. Q. (2020). Effect of Chinese medicine on No or slow reflow after percutaneous coronary intervention in myocardial infarction patients: A systematic review and meta-analysis. Chin. J. Integr. Med. 26 (3), 227–234. 10.1007/s11655-019-2703-9 31093877

[B134] Zhang Y. Wang Y. Wang L. Qin Y. Chen T. Han W (2012). Effects of Rehmannia glutinosa oligosaccharide on human adipose-derived mesenchymal stem cells *in vitro* . Life Sci. 91, 1323–1327. 10.1016/j.lfs.2012.10.015 23123441

[B135] Zhao L. Chen S. Yang P. Cao H. Li L. (2019). The role of mesenchymal stem cells in hematopoietic stem cell transplantation: Prevention and treatment of graft-versus-host disease. Stem Cell. Res. Ther. 10 (1), 182. 10.1186/s13287-019-1287-9 31227011 PMC6588914

[B136] Zhao T. T. Liu J. J. Zhu J. Li H. Wang Y. C. Zhao Y. (2022). SDF-1/CXCR4-Mediated stem cell mobilization involved in cardioprotective effects of electroacupuncture on mouse with myocardial infarction. Oxid. Med. Cell. Longev. 2022, 4455183. 10.1155/2022/4455183 35982734 PMC9381195

[B137] Zheng H. Liu C. Ou Y. Zhang Y. Fu X. (2013). Total saponins of Panax notoginseng enhance VEGF and relative receptors signals and promote angiogenesis derived from rat bone marrow mesenchymal stem cells. J. Ethnopharmacol. 147 (3), 595–602. 10.1016/j.jep.2013.03.043 23545458

[B138] Zhou J. Zhang J. Wu L. Zhu F. Xu H. (2020). Tetramethylpyrazine/ligustrazine can improve the survival rate of adipose-derived stem cell transplantation. Ann. Plast. Surg. 84 (3), 328–333. 10.1097/SAP.0000000000002146 31972572

[B139] Zhu L. Liu Y. J. Shen H. Gu P. Q. Zhang L. (2017). Astragalus and baicalein regulate inflammation of mesenchymal stem cells (MSCs) by the mitogen-activated protein kinase (MAPK)/ERK pathway. Med. Sci. Monit. 23, 3209–3216. 10.12659/msm.902441 28667247 PMC5507801

