# Supplementary material for: Intervention effects of traditional Chinese medicine on stem cell therapy of myocardial infarction
Source: Front Pharmacol. 2022 Oct 18;13:1013740. doi: 10.3389/fphar.2022.1013740 (PMC9622800; doi:10.3389/fphar.2022.1013740)
Supplement: Supplementary file 1 [file Table1.docx]

**Table S1 Compositions of TCM prescription**

| **Prescription** | **Composition** |
| --- | --- |
| Guanxin Danshen formulation  (冠心丹参方) | *Salvia miltiorrhiza*Bunge [Lamiaceae] (Dan Shen), *Panax notoginseng* (Burkill) F.H. Chen [Araliaceae] (San Qi), *Dalbergia odorifera* T.C. Chen [Fabaceae] (Jiang Xiang) |
| Taohong siwu decoction  ( 桃红四物汤) | *Prunus persica* (L.) Batsch [Rosaceae] (Tao Ren), *Carthamus tinctorius L.* [Asteraceae] (Hong Hua), *Angelica sinensis* (Oliv.) Diels [Apiaceae] (Dang Gui), *Paeonia lactiflora* Pall. [Paeoniaceae] (Shao Yao), *Ligusticum striatum*DC. [Apiaceae] (Chuan Xiong), *Rehmannia glutinosa* (Gaertn.) DC. [Orobanchaceae] (Di Huang) |
| Xuefu Zhuyu Decoction  (血府逐瘀汤) | *Angelica sinensis* (Oliv.) Diels [Apiaceae] (Dang Gui), *Rehmannia glutinosa* (Gaertn.) DC. [Orobanchaceae] (Di Huang), *Prunus persica* (L.) Batsch [Rosaceae] (Tao Ren), *Carthamus tinctorius L*. [Asteraceae] (Hong Hua), Fructus Aurantii Immaturus (Zhi Shi), *Paeonia lactiflora* Pall. [Paeoniaceae] (Shao Yao), *Bupleurum falcatum*L. [Apiaceae] (Chai Hu), *Glycyrrhiza uralensis* Fisch. ex DC. [Fabaceae] (Gan Cao), *Platycodon grandiflorus* (Jacq.) A. DC. [Campanulaceae] (Jie Geng), *Ligusticum striatum*DC. [Apiaceae] (Chuan Xiong), *Cyathula officinalis*K.C. Kuan [Amaranthaceae] (Niu Xi) |
| Shuangxinfang  (双心方) | *Salvia miltiorrhiza*Bunge [Lamiaceae] (Dan Shen), *Ligusticum striatum*DC. [Apiaceae] (Chuan Xiong), *Ziziphus jujuba*Mill. [Rhamnaceae] (Da Zao), *Lilium lancifolium*Thunb. [Liliaceae] (Bai He) |
| Shuanglong Formula  (双龙方) | *Panax ginseng* C.A. Mey. [Araliaceae] (Ren Shen), *Salvia miltiorrhiza*Bunge [Lamiaceae] (Dan Shen) |
| Tongxinluo  (通心络) | *Panax ginseng* C.A.Mey. [Araliaceae] (Ren Shen), Buthus Martensi (Quan Xie), Hirudo medicinalis (Shi Zhi), Eupolyphaga seu steleophage (Tu Bie Chong), Scolopendra subspinipes (Wu Gong), Periostracum cicadae (Chan Tui), *Paeonia lactiflora Pall.* [Paeoniaceae] (Shao Yao), *Ziziphus jujuba Mill.* [Rhamnaceae] (Da Zao), *Dalbergia odorifera*T.C.Chen [Fabaceae] (Jiang Xiang), *Santalum album* L. [Santalaceae] (Tan Xiang), *Cinnamomum camphora* (L.) J.Presl [Lauraceae] (Bing Pian) |
| Danhong injection  (丹红注射液) | *Salvia miltiorrhiza*Bunge [Lamiaceae] (Dan Shen), *Carthamus tinctorius* L. [Asteraceae] (Hong Hua) |
| Xuesaitong Injection  (血塞通注射液 ) | *Panax notoginseng* (Burkill) F.H. Chen [Araliaceae] (San Qi) |
| Si-wu decoction  (SDE, 四物汤) | *Ligusticum striatum*DC. [Apiaceae] (Chuan Xiong), *Angelica sinensis* (Oliv.) Diels [Apiaceae] (Dang Gui), *Paeonia lactiflora*Pall. [Paeoniaceae] (Chi Shao), *Rehmannia glutinosa* (Gaertn.) DC. [Orobanchaceae] (Di Huang) |
| Gu Ben Pei Yuan San  (固本培元散) | *Panax ginseng* C.A.Mey. [Araliaceae] (Ren Shen), Cervi Cornu Pantorichum. (Ru Long), Placenta Hominis (Zi He Che), Trogopterus dung (Wu Ling Zhi), *Panax notoginseng* (Burkill) F.H. Chen [Araliaceae] (San Qi), G. gecko Linnaeus tails (Ge Jie) |
